# Ionic liquid-loaded triazine-based magnetic nanoparticles for promoting multicomponent reaction

**DOI:** 10.1038/s41598-022-26235-6

**Published:** 2022-12-23

**Authors:** Kosar Kafshdarzadeh, Masoume Malmir, Zahra Amiri, Majid M. Heravi

**Affiliations:** Department of Organic Chemistry, Faculty of Chemistry, Alzahra University, Vanak, Tehran, Iran

**Keywords:** Catalysis, Catalyst synthesis, Catalytic mechanisms, Heterogeneous catalysis

## Abstract

A novel hybrid magnetic ionic-liquid as a heterogeneous catalyst was synthesized by hybridization of imidazolium based-ionic liquid onto the nitrogen rich magnetic nanocomposite. The resulting catalyst (n-Fe_3_O_4_@SiO_2_-TA-SO_3_H IL) has two advantages besides recyclability: (i) high capacity of functional-SO_3_H group with imidazolium-IL cation for promoting symmetric and asymmetric Hantzsch reaction and (ii) easy recovery. Caused by the polymeric and magnetic nature of the n-Fe_3_O_4_@SiO_2_-TA-SO_3_H IL, large quantities of acidic groups were bound to the n-Fe_3_O_4_@SiO_2_-TA surface, which reduced the catalyst mass applied to the catalytic reaction. Moreover, superior catalytic performance and outstanding recyclability of n-Fe_3_O_4_@SiO_2_-TA-SO_3_H IL in mild condition make this method a green pathway for manufacture of satisfactory chemicals.

## Introduction

Nowadays, chemistry is motivated on developing effective strategies for important chemical transformations that are used in environmentally friendly and sustainable conditions. However, it is vital to consider the principles of green chemistry, with particular regard to the methods that are moving towards a sustainable chemical industry^1^. For this purpose, there have been major worries about minimization of waste as well as sustainability, because these are modern and important issues and involve environmental features^2^.

**Figure 1:**
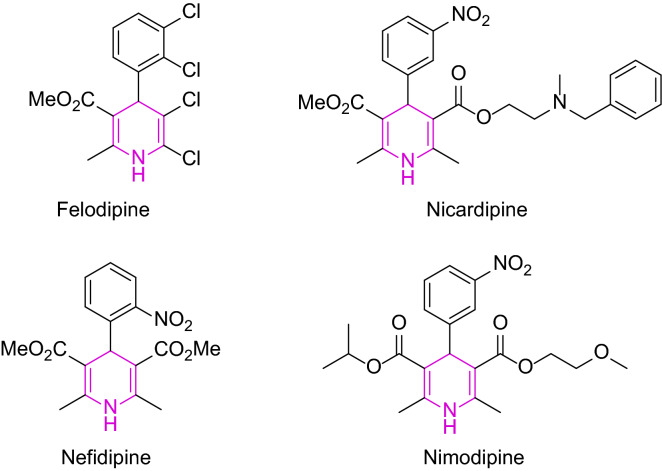
1,4-DHP derivatives employed as clinical drugs.

Multi-component reactions (MCRs) which use several reagents to provide corresponding products having structure of all beginning materials in one set and one-pot of stable conditions are some strong productivity examples^3^. Among, the Hantzsch reaction (HnR) which generates 1,4-dihydropyridines (1,4-DHP) products is one of the most distinguished MCRs due to outstanding biological profile of 1,4-DHP^4^. There are many examples in pharmaceuticals area of 1,4-DHP as a calcium channel blockers, for instance, Felodipine, Nicardipine, Nifedipine, and Nimodipine (Fig. 1). Moreover, other flexible biological sketches of 1,4-DHP such as adenosine receptor antagonism^5^, anticonvulsant activity^6^, radioprotective activity^7^, inhibition, and sirtuin activation^8^, etc. have also been reported. Hence, 1,4-DHP are recognized as important substances in chemical, pharmaceutical, and biological syntheses.

In this regard, many efforts are now being made to link the some benefits for performing MCRs^9^, by ionic liquids (ILs)^10^. Recently, ILs have been pronounced as an eco-friendly reaction medium^11^, employment of their in the chemical industry and also organic transformations, specially MCRs, has become really remarkable^12,13^.

Nanoparticles (NPs) as a catalytic sites furnish the advantage of increased surface area as well as reaction rate^14,15^. One particularly appropriate and significant group of NPs is magnetic nanoparticles (MNPs). MNPs could be linked to a series of organic and inorganic materials^16,17^. One of the prominent features of MNPs is their strong magnetic moments and separation by an external magnetic field. These attributes are of use for a several of probable applications in the field of biology and medicine^18–22^. Furthermore, MNPs can serve as a very useful catalyst backer that allows the catalyst to be immobilized and magnetically recovered^23^. Recently, remarkable consideration has been focused on Magnetic Ionic Liquids (MILs). MILs with the general properties of ILs and metals in their structure, can have various applications, including optical/ photophysical and catalytic properties. One of the first examples was the case study of Hayashi et al. in 2004^24,25^. In this regard, several green MCR protocols^26–28^ for the synthesis of 1,8-dioxo-decahydroacridine (1,8-DOXDHA) and 1,4-dyhdropyridine (DHP) derivatives using Fe_3_O_4_@SiO_2_-based nanocatalysts were reported^29–34^.

Following of our study on the design and preparation of heterogeneous catalysts in this line development of environmentally benign procedures to synthesis of chemicals, recently, we focused on the utility of magnetic and *N*-doped heterogeneous catalysts for developing some organic transformations^35–39^. In this line, we are demonstrating efficient catalysts by preparing and decorating of MNPs with *N*-doped functionalities and ILs, n-Fe_3_O_4_@SiO_2_-TA-SO_3_H IL. In this regard, we investigated the symmetric HnR between differently substituted aromatic aldehydes, ammonium acetate, dimedone or ethyl acetoacetate for the formation of some 1,8-DOXDHA and 1,4-DHP. Moreover, some polyhydroquinolines (PHQ) were synthesized via four-component reaction of various substituted ammonium acetate, aldehydes, ethyl-acetoacetate, and dimedone in excellent yields.

## Result and discussion

### Catalyst characterization

**Figure 2:**
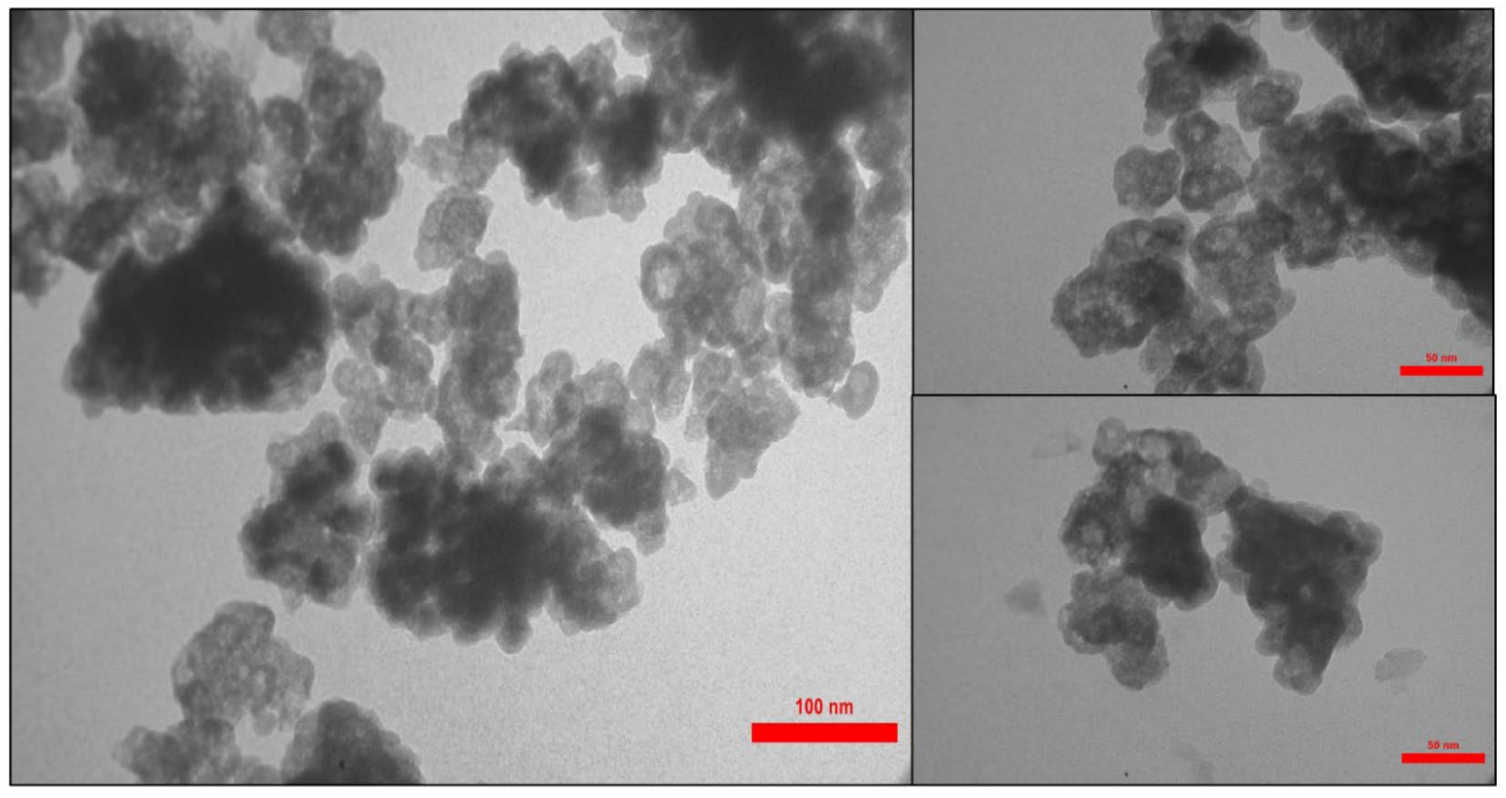
TEM images of n-Fe_3_O_4_@SiO_2_-TA-SO_3_H IL.

First, morphological characteristics of the n-Fe_3_O_4_@SiO_2_-TA-SO_3_H IL and ILs including shape and size envisioned by TEM, SEM/EDX and elemental mapping analysis. TEM images displayed black-spherical morphology with some amount of agglomeration (Fig. 2). As shown in Fig. 2, n-Fe_3_O_4_ are surrounded with gray coverage, confirming the successful incorporation of ILs and organic layer in the structure of the n-Fe_3_O_4_@SiO_2_-TA-SO_3_H IL. Moreover, the mean diameter of n-Fe_3_O_4_ was found to be ∼17.8 nm which is almost consistent with result obtained from Debyee-Scherrer Equation (17.2 nm). Nevertheless, this manner resulted in successful decoration of n-Fe_3_O_4_ with ILs and organic linkage.

Next, to further investigation of the catalyst formation, the structure of ILs and n-Fe_3_O_4_@SiO_2_-TA-SO_3_H IL were analyzed via SEM/EDS, Fig. S2. As exhibited in Fig. S2a, ILs has a thin-layer and delicate structure. Moreover, in agreement with the layer-like morphology of ILs, it can be clear that the spherical n-Fe_3_O_4_ and ILs were placed next to each other with minimal adhesion (Fig. S2c). These results also confirmed that the multi-stage functionalization on n-Fe_3_O_4_ and decoration with ILs was successful. On the other hand, the EDX analysis contains sulfur, oxygen, carbon and nitrogen elements, representing the successful formation of ILs (Fig. S2b)_._ The EDX image of n-Fe_3_O_4_@SiO_2_-TA-SO_3_H IL exhibits all available elements including, sulfur, nitrogen, carbon, silicon, oxygen, and iron elements (Fig. S2d). In Fig. S3, the elemental-mapping analysis (EMA) of ILs is described, which confirms the presence of iron, oxygen, nitrogen, and sulfur based on the successful synthesis of ILs. Moreover, as demonstrated in Fig. S4, C, N, S, Fe, Si, and O atoms were dispersed practically uniform, confirming ILs have been well synthesized and well-coated by n-Fe_3_O_4._

The typical FTIR spectra of the n-Fe_3_O_4_, n-Fe_3_O_4_@SiO_2_, ILs, n-Fe_3_O_4_@SiO_2_-NH_2_, n-Fe_3_O_4_@SiO_2_-TA, and n-Fe_3_O_4_@SiO_2_-TA-SO_3_H IL are exhibited in Fig. S5a–f, respectively. The bare n-Fe_3_O_4_ (Fig. S5a) showed two major peaks around 3400 and 584 cm^−1^ causing from the stretching vibrations of Fe–O and hydroxyl groups^40^. In Fig. S5b, clearly characteristic bands of silica shell at 1103 cm^−1^ and two bands at 990 and 830 cm^−1^ were observed, which can be allotted to Si–O and Si–O–Si stretching vibrations, respectively. It should be noted that Fig. S5b,c are almost similar, which can be related to the low percentage of functionalization of n-Fe_3_O_4_@SiO_2_ with -NH_2_ groups. In Fig. S5d, the band at 1654 cm^−1^ is very observable that confirms the presence of C=N groups and triazine ring in the structure of Fe_3_O_4_@SiO_2_-TA. Moreover, the band at 2972 cm^−1^ can be assigned to –CH_2_ stretching. The synthesis of ILs was also confirmed by detecting several peaks at 1053, 1162, 1651, 2965 and 3125 cm^−1^, Which can assigned to S–O, S=O, C=N, –CH_2_, and hydroxyl groups, respectively (Fig. S5e). In Fig. S5f, the observed peak at 1654 cm^−1^ is related to the imine (C=N) functionality and can confirm the conjugation of n-Fe_3_O_4_@SiO_2_-TA with ILs^41^. In addition, the FTIR of 1*H*-imidazole, 1,4-butane sultone and 2,4,6-trichloro-1,3,5-triazine are given in the supplementary as a Fig. S6.

Next, the formation and structure of n-Fe_3_O_4_@SiO_2_-TA-SO_3_H IL was also studied and verified by the XRD pattern. As presented in Fig. S7 and confirming to XRD pattern of bare n-Fe_3_O_4_ (Fig. S7), the characteristic bands of n-Fe_3_O_4_ were appeared at 2*Ɵ* = 30.12° (220), 35° (311), 43.17° (400), 53.58° (422), 57.10° (511), 62.65° (440), and 74.28° (622) (labelled as *) with the cubic inverse spinel structure (JCPDS card no. 39–1346)^42^. It is worth mentioning that the characteristic bands around 2*θ* = 25° is related to SiO_2_.

In Supplementary Fig. S8, the thermal stability of the (a) n-Fe_3_O_4_, (b) n-Fe_3_O_4_@SiO_2_, (c) ILs, (d) n-Fe_3_O_4_@SiO_2_-NH_2_, (e) n-Fe_3_O_4_@SiO_2_-TA, and (f) n-Fe_3_O_4_@SiO_2_-TA-SO_3_H IL are demonstrated. As shown in Fig. S8a, n-Fe_3_O_4_ possessed high thermal stability and exhibited only the weight loss below 200 °C that is representative of loss of water. Fig. S8b shows a major loss due to the elimination of water and –OH groups that the amount of silica on the n-Fe_3_O_4_ surface was calculated to be 5%. According to Fig. S8c, two weight losses for ILs were detected; the first is related to the loss of moisture and water at a temperature of less than 150 °C and the second is related to the rupture of organic bonds in ILs at less than 550 °C. Moreover, the weight losses of about 2.7% and 3.9% can be due to the decomposition of APTES and organic linkage in the structure of n-Fe_3_O_4_@SiO_2_-TA-SO_3_H IL (Fig. S8d,e). By the comparison of n-Fe_3_O_4_@SiO_2_-TA-SO_3_H IL, n-Fe_3_O_4_@SiO_2_-TA with that of ILs, three steps degradation were observed, including loss of hydroxyl groups and water at 175 °C and the major weight loss about 20.22 wt% between 230 and 600 °C is attributed to ILs decomposition and degradation of organic–inorganic linkage (Fig. S8f).

To investigate the textural properties of the n-Fe_3_O_4_@SiO_2_-TA-SO_3_H IL, the N_2_ adsorption–desorption isotherm and BJH plot were recorded and represented in Supplementary Fig. S9. As shown, the shape of n-Fe_3_O_4_@SiO_2_-TA-SO_3_H IL exhibited type II isotherm with H3 hysteresis loops^43^. The specific surface area and pore volume of the catalyst was calculated to be 8.9 m^2^g^−1^ and 0.068 cm^3^g^−1^, respectively, which was lower than that of n-Fe_3_O_4_ (124 m^2^g^−1^ and 0.073 cm^3^g^−1^). These results can indicate all pores in n-Fe_3_O_4_ are well-covered. Moreover, the pore-size distribution curve of n-Fe_3_O_4_@SiO_2_-TA-SO_3_H IL was obtained by the BJH method using the pore volumes in the measurement of N_2_ desorption isotherms. As is evident from pore-size distribution result of n-Fe_3_O_4_@SiO_2_-TA-SO_3_H IL, the major types of pores with micropores (7.4–9 nm) was clear.

**Figure 3:**
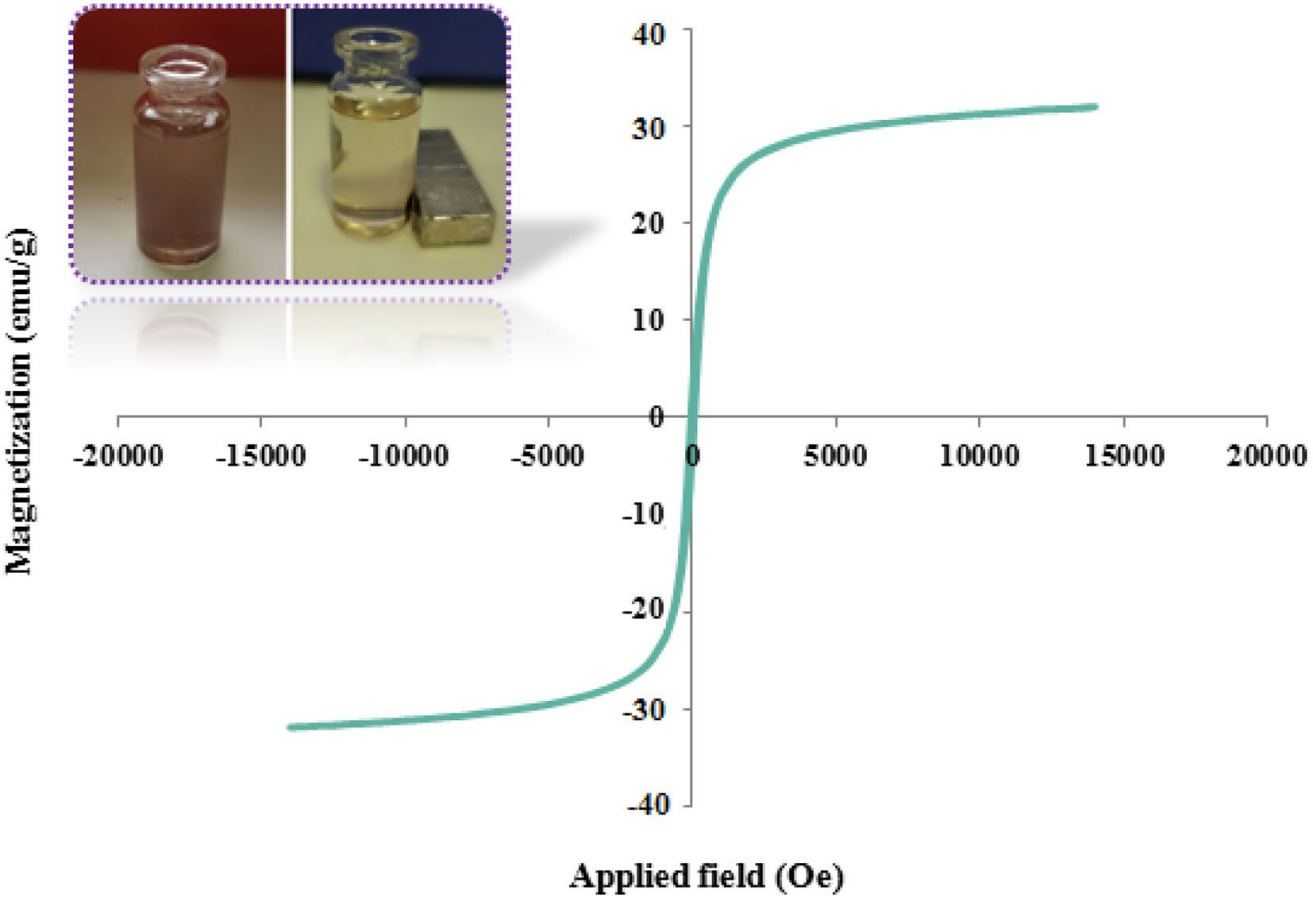
VSM analysis of n-Fe_3_O_4_@SiO_2_-TA-SO_3_H IL.

To elucidate whether decoration of the n-Fe_3_O_4_ surface with SiO_2_, ILs, and organic linkage can alter the magnetic property, n-Fe_3_O_4_@SiO_2_-TA-SO_3_H IL was studied at room temperature using VSM, and its magnetic features compared with n-Fe_3_O_4_ (Fig. 3). As can be seen, the *M*_s_ values of n-Fe_3_O_4_@SiO_2_-TA-SO_3_H IL was about 31 emu g^-1^ that this value was much lower than that of n-Fe_3_O_4_ (101 emu g^−1^)^44^. These results indicated that incorporation of nonmagnetic component can markedly reduce the magnetic property of n-Fe_3_O_4_. However, the hysteresis loops of both n-Fe_3_O_4_@SiO_2_-TA-SO_3_H IL exhibited a super paramagnetic behavior with their dispersion stability in solution, without aggregation.

### Investigation of the catalytic activity

To consider the catalytic activity of this catalytic system, the n-Fe_3_O_4_@SiO_2_-TA-SO_3_H IL was employed as a recyclable catalyst in symmetry HnR. Initially, the reaction of 4-nitrobenzaldehyde (**1b**) (1 mmol), ammonium acetate (**2**) (1 mmol), and dimedone (**3a**) (2 mmol) was selected as a model substrate. To find an optimum conditions for the symmetric HnR, the effect of different parameters for example type of solvents, reaction temperature, and catalyst loading were investigated in a model reaction (Table S1). In the next step, the model reaction was conducted at various temperatures (r.t., 50 °C and reflux). The results demonstrated that the HnR under refluxing condition leads to higher efficiency for the expected product (Table S1, entries 1–3). Then, various reaction mediums for instance H_2_O, EtOH, EtOH/H_2_O, DMF, THF, MeCN, CHCl_3_, MeOH, CH_2_Cl_2_, and toluene were applied (Table S1, entries 3–12). As a result, H_2_O was selected as the optimal solvent. In order to achieve and the optimized catalyst loading, different amounts of the n-Fe_3_O_4_@SiO_2_-TA-SO_3_H IL (0, 8, 16 and 32 mg) were examined (Table S1, entries 3, 13, 15). As tabulated, 16 mg of the n-Fe_3_O_4_@SiO_2_-TA-SO_3_H IL was the best. Importantly, an appropriate result was accomplished by employing H_2_O as a suitable reaction media using n-Fe_3_O_4_@SiO_2_-TA-SO_3_H IL (16 mg) under reflux condition. In an effort to generalize the optimal condition and assessing the appropriate efficiency and performance of n-Fe_3_O_4_@SiO_2_-TA-SO_3_H IL, a wide-range of electron-releasing and electron-withdrawing substitutions-aldehydes, 1,3-diketone (dimedone) or β-ketoester (ethyl-acetoacetate), and ammonium acetate were used (Table 1, entries 1–16). A variety of substituted 1,8-DOXDHA and 1,4-DHP were fabricated using employing n-Fe_3_O_4_@SiO_2_-TA-SO_3_H IL (16 mg) under refluxing in water (Table 1). As tabulated, the isolated and expected products were achieved in excellent yields and short times.

**Table 1:** One-pot synthesis of 1,8-DOXDHA and 1,4-DHP^a^.


Entry	R	1,3-diketone	Product	Time (h:min)	TON^b^	TOF^c^ (min^−1^)	Yield^d^ (%)	Conv	Melting point (°C)	Melting point (°C)
									Observed	Reported
1	H	**3a**	**4a**	00:25	9.0	0.36	90	95	289–291	290–293^50^
2	4-NO_2_	**3a**	**4b**	00:08	9.7	1.21	97	99	285–287	285–287^51^
3	3-NO_2_	**3a**	**4c**	00:15	9.0	0.60	90	95	294–297	295–296^26^
4	2-NO_2_	**3a**	**4d**	00:05	9.5	1.90	95	97	289–292	289–292^50^
5	4-Cl	**3a**	**4e**	00:25	9.0	0.36	90	93	304–306	303–305^52^
6	4-Br	**3a**	**4f**	00:20	9.2	0.46	92	95	240–243	240–242^53^
7	4-OMe	**3a**	**4g**	00:35	8.3	0.24	83	85	267–269	269–271^54^
8	4-Me	**3a**	**4h**	00:25	8.5	0.34	85	90	278–280	279–281^55^
9	2-Br	**3a**	**4i**	00:08	9.2	1.15	92	95	252–254	252–254^56^
10	H	**3b**	**5a**	00:50	8.8	0.18	88	92	174–176	176–178^57^
11	4-NO_2_	**3b**	**5b**	00:40	7.0	0.17	70	80	122–124	124–127^58^
12	3-NO_2_	**3b**	**5c**	01:00	5.5	0.09	55	65	162–166	164–165^59^
13	4-Cl	**3b**	**5d**	01:00	7.0	0.12	70	75	145–148	145–147^27^
14	4-Br	**3b**	**5e**	00:45	9.2	0.20	92	95	158–160	158–160^60^
15	4-OMe	**3b**	**5f**	01:05	7.8	0.12	78	85	154–156	156–158^61^
16	4-Me	**3b**	**5g**	00:50	8.5	0.17	85	90	136–138	136–138^62^

^a^Reaction conditions: aldehydes (1.0 mmol), 1,3-diketone (2.0 mmol), ammonium acetate (1.0 mmol), n-Fe_3_O_4_@SiO_2_-TA-SO_3_H IL in H_2_O (5.0 mL) under reflux.

^b^TON = Yield (%)/Catalyst amount (mol%).

^c^TOF = TON/time (min).

^d^Isolated yield.

Next, in order to achieve the optimal reaction conditions for the asymmetric HnR, the effect of different parameters for example type of solvents, reaction temperature, and catalyst loading were investigated in a model reaction containing 4-nitrobenzaldehyde (**1b**) (1 mmol), ammonium acetate (**2**) (1 mmol), dimedone (**3a**) (1 mmol), and ethyl acetoacetate (**3b**) (1 mmol) (Table S2). At first, the model reaction was carried out in different reaction mediums for instance H_2_O, EtOH, EtOH/H_2_O, MeCN, and MeOH (Table S2, entries 1–5). As a result, EtOH as the best solvent for producing expected product with high yield. Then, the model reaction was examined by different temperatures (Table S2, entries 2, 6 and 7) and the refluxing conditions was selected as an appropriate temperature. Finally, for the sake of catalyst loading, various amounts of n-Fe_3_O_4_@SiO_2_-TA-SO_3_H IL (0, 8, 16, and 32 mg) were investigated (Table S2, entries 2, 8–10). As tabulated, 16 mg of the n-Fe_3_O_4_@SiO_2_-TA-SO_3_H IL was the best for asymmetry HnR. As a result, the best consequence was achieved by using 16 mg of n-Fe_3_O_4_@SiO_2_-TA-SO_3_H IL as catalyst in EtOH under reflux conditions. Beside of catalytic performance of n-Fe_3_O_4_@SiO_2_-TA-SO_3_H IL for development and generalization of 1,8-DOXDHA and 1,4-DHP derivatives trough symmetric HnR, its possibility were considered for PHQ synthesis. Regarding, a wide-range of electron-releasing and electron-withdrawing substitutions-aldehydes reacted with ammonium acetate, dimedone, and ethyl acetoacetate under optimized condition. Worthy to mention that, several substituted-PHQ (**6a–f**) were synthesized in short reaction times with high yields (Table 2).

**Table 2:** One-pot synthesis of PHQ derivatives^a^.


Entry	R	Product	Time (h:min)	TON^b^	TOF^c^ (min^−1^)	Yield^d^ (%)	Conv	Melting point (°C)	Melting point (°C)
								Observed	Reported
1	H	**6a**	01:30	8.9	0.10	89	92	202–204	203–204^63^
2	4-NO_2_	**6b**	00:40	9.0	0.22	90	95	239–241	236–237^28^
3	2-OH	**6c**	00:10	9.9	0.99	96	99	208–210	208–211^64^
4	4-OMe	**6d**	01:00	9.0	0.15	85	90	254–257	254–256^65^
5	4-Cl	**6e**	00:45	9.5	0.21	92	95	248–250	248–250^66^
6	4-Br	**6f**	01:10	9.0	0.13	82	90	250–252	252–254^67^

^a^Reaction conditions: aldehydes (1.0 mmol), dimedone (1.0 mmol), ethyl acetoacetate (1.0 mmol), ammonium acetate (1.0 mmol), n-Fe_3_O_4_@SiO_2_-TA-SO_3_H IL in EtOH (5.0 mL) under reflux.

^b^TON = Yield (%)/Catalyst amount (mol%).

^c^TOF = TON/time (min).

^d^Isolated yield.

A reasonable mechanism for the present HnR is displayed in Supplementary Fig. S1. In this reaction, n-Fe_3_O_4_@SiO_2_-TA-SO_3_H IL can activate reactants containing –C=O groups (aldehydes, dimedone, and 1,3-diketon or β-ketoester) and intermediates. At first, an intermediate (**I**) was produced by the reaction of an activated aldehyde **1ꞌ** and dimedone **3a'** or 1,3-ketoesters **3bꞌ** using of n-Fe_3_O_4_@SiO_2_-TA-SO_3_H IL. Later, an intermediate II was generated by adding the next mole of **3aꞌ** or **3bꞌ** to (**I**). Afterwards, with the addition of ammonium acetate and its interaction with carbonyl of intermediate (**II**), an intermediate (**III**) was obtained. In the last step, corresponding products **(4a–i** or **5a–g** or **6a–f**) were achieved by an imine-enamine tautomerization.

### Catalyst recyclability

**Figure 4:**
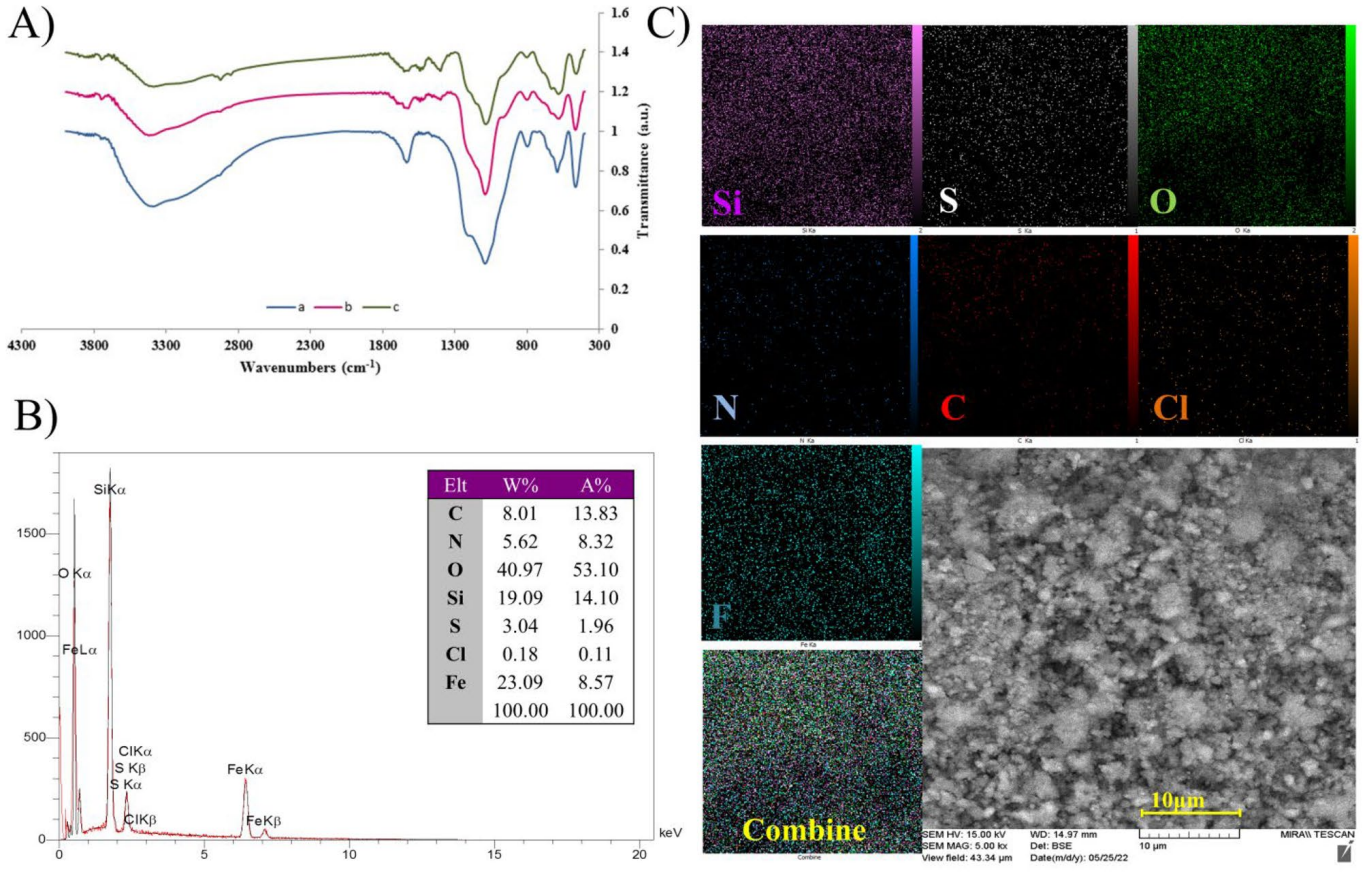
(**A**) The FT-IR spectra of (a) n-Fe_3_O_4_@SiO_2_-TA-SO_3_H IL, (b) n-Fe_3_O_4_@SiO_2_-TA-SO_3_H IL after one time reusing in the reaction, (c) n-Fe_3_O_4_@SiO_2_-TA-SO_3_H IL after eight times reusing in the reaction; (**B**) The EDX analysis of reused n-Fe_3_O_4_@SiO_2_-TA-SO_3_H IL; (**C**) The elemental mapping analysis of reused n-Fe_3_O_4_@SiO_2_-TA-SO_3_H IL.

The recyclability and reusability of n-Fe_3_O_4_@SiO_2_-TA-SO_3_H IL were evaluated in symmetric and asymmetric HnR. Intentionally, the reaction between 4-bromobenzaldehyde, ammonium acetate, and ethyl acetoacetate using n-Fe_3_O_4_@SiO_2_-TA-SO_3_H IL in H_2_O under reflux condition for diethyl 4-(4-bromophenyl)-2,6-dimethyl-1,4-dihydropyridine-3,5-dicarboxylate (**5e**) preparation was accomplished. After the first run, the n-Fe_3_O_4_@SiO_2_-TA-SO_3_H IL was separated by an external magnet, washed with warm EtOH. Next, the reused n-Fe_3_O_4_@SiO_2_-TA-SO_3_H IL was employed for next run under above-mentioned conditions. This exploration demonstrated that this catalyst can be re-covered as a minimum eight runs with minimal losing of catalytic activity (Fig. S10). In addition, we examined recyclability of n-Fe_3_O_4_@SiO_2_-TA-SO_3_H IL in asymmetric HnR. To this goal, the reaction of 2-hydroxybenzaldehyde, ammonium acetate, dimedone, and ethyl-acetoacetate using n-Fe_3_O_4_@SiO_2_-TA-SO_3_H IL in EtOH under reflux condition for ethyl 4-(2-hydroxyphenyl)-2,7,7-trimethyl-5-oxo-1,4,5,6,7,8-hexahydroquinoline-3-carboxylate (**6c**) preparation was performed. After first run, the catalyst was separated by a magnet, washed with warm EtOH. Next, the reused catalyst was applied in next run under the above-mentioned. This investigation demonstrated that this catalyst could be recovered and reused at least eight times without significant loss of its catalytic activity (Fig. S10). Worthy to mention that Fig. 4A–C shows the comparison between FTIR of the n-Fe_3_O_4_@SiO_2_-TA-SO_3_H IL, Fe_3_O_4_@SiO_2_-TA-SO_3_H IL after one time reusing in HnR and Fe_3_O_4_@SiO_2_-TA-SO_3_H IL after eight times reusing in the HnR and EDX/ elemental mapping analysis. It turned out, the FTIR spectrum of the reused Fe_3_O_4_@SiO_2_-TA-SO_3_H IL was similar to that of the fresh one and no characteristic band of Fe_3_O_4_@SiO_2_-TA-SO_3_H IL has been disappeared upon reusing (Fig. 4A). Hence, Fe_3_O_4_@SiO_2_-TA-SO_3_H IL benefits from good stability. Moreover, the results of EDX and elemental mapping analysis also confirm the presence of elements, but the reason for the slight decrease in catalytic activity can be due to the polar interaction of active acidic sites with the aqueous reaction medium and subsequent washing which causes leaching of proton to the reaction medium and reduction of acidic sites (Fig. 4B,C).

After the oversimplification process of the n-Fe_3_O_4_@SiO_2_-TA-SO_3_H IL in symmetry synthesis of 1,8-DOXDHA and 1,4-DHP (Table 3, entries 1–6) and asymmetry synthesis of PHQ derivatives, its remarkable performance compared with other prior literatures (Table 3, entries 17–19) and several control catalysts including, n-Fe_3_O_4_, n-Fe_3_O_4_@SiO_2_, n-Fe_3_O_4_@SiO_2_-NH_2_, and n-Fe_3_O_4_@SiO_2_-TA (Table 3, entries 7–10 and 20–23). As observed, the comparative results based on various parameters involving the temperature, solvent, catalyst, time, and yield of products are summarized in Table 3. As tabulated, all reported literatures are supplemented with some drawbacks for instance, employing costly and weak catalyst, harsh conditions, and hard work-up procedures. Nevertheless, the privileges of this catalytic system compared to recent reported include high catalytic capacity, significant magnetic property, mild conditions, high yield percentage of isolated products that are formed in shorter time and mild condition. Moreover, based on the analytical and experimental results, the amount of acidic protons in the catalyst is about 10 mol%, which is the active site to promote this reaction. Hence, this result were compared to other traditional acid catalysts (Table 3, entries 11–16 and 24–29), which the amount of acid in the structure of ionic liquid compared to other acidic catalysts is not only suitable but also safe and has no corrosive effect and can be easily separated. Finally, these findings indicated that the enhanced magnetic catalytic activity of n-Fe_3_O_4_@SiO_2_-TA-SO_3_H IL could be attributed to the synergistic effect of IL and n-Fe_3_O_4_@SiO_2_-TA.

**Table 3:** Comparison of the efficiency of n-Fe_3_O_4_@SiO_2_-TA-SO_3_H IL with reported catalysts for the preparation of (**4b**) via symmetric HnR^a^ and (**6c**) via asymmetric HnR^b^.

Entry	Catalyst (Amount of catalyst)	Solvent	Temperature(°C)	Time (h:min)	Yield^c^ (%)	Ref
1	HY-Zeolite (0.1 g)	EtOH	Reflux	03:00	79	^68^
2	choline chloride (5 mol%)	EtOH	120	03:00	83	^69^
3	L-proline (15 mol%)	H_2_O	Reflux	02:10	92	^70^
4	Silica-supported Preyssler nanoparticles (SPNP) (0.03 mmol)	H_2_O	Reflux	02:00	90	^71^
5	SD-OSO_3_H (0.05 g)	EtOH	Reflux	00:35	90	^72^
6	NiFe_2_O_4_@SiO_2_‐FHS (0.025 g)	S.F	80	00:15	91	^53^
7	n-Fe_3_O_4_ (10 mol%)	H_2_O	Reflux	00:08	35	This study
8	n-Fe_3_O_4_@SiO_2_ (10 mol%)	H_2_O	Reflux	00:08	40	This study
9	n-Fe_3_O_4_@SiO_2_-NH_2_ (10 mol%)	H_2_O	Reflux	00:08	45	This study
10	n-Fe_3_O_4_@SiO_2_-TA (10 mol%)	H_2_O	Reflux	00:08	45	This study
11	CH_3_COOH (10 mol%)	H_2_O	Reflux	00:08	Trace	This study
12	HNO_3_ (10 mol%)	H_2_O	Reflux	00:08	10	This study
13	H_2_SO_4_ (10 mol%)	H_2_O	Reflux	00:08	20	This study
14	HCl (10 mol%)	H_2_O	Reflux	00:08	10	This study
15	3-(*n*-butanesulfonate)-imidazole (10 mol%)	H_2_O	Reflux	00:08	90	This study
16	n-Fe_3_O_4_@SiO_2_-TA-SO_3_H IL (10 mol%)	H_2_O	Reflux	00:08	97	This study
17	Cu-NP/C (0.02 g)	EtOH	Reflux	02:00	87	^73^
18	[Msim]Cl (10 mol%)	EtOH	80	03:30	90	^74^
19	Fe_2_O_3_@HAp@Melamine (0.15 g)	S.F	80	00:15	94	^75^
20	n-Fe_3_O_4_ (10 mol%)	EtOH	Reflux	00:10	30	This study
21	n-Fe_3_O_4_@SiO_2_ (10 mol%)	EtOH	Reflux	00:10	35	This study
22	n-Fe_3_O_4_@SiO_2_-NH_2_ (10 mol%)	EtOH	Reflux	00:10	35	This study
23	n-Fe_3_O_4_@SiO_2_-TA (10 mol%)	EtOH	Reflux	00:10	40	This study
24	CH_3_COOH (10 mol%)	EtOH	Reflux	00:10	Trace	This study
25	HNO_3_ (10 mol%)	EtOH	Reflux	00:10	15	This study
26	H_2_SO_4_ (10 mol%)	EtOH	Reflux	00:10	25	This study
27	HCl (10 mol%)	EtOH	Reflux	00:10	20	This study
28	3-(*n*-butanesulfonate)-imidazole (10 mol%)	EtOH	Reflux	00:10	88	This study
29	n-Fe_3_O_4_@SiO_2_-TA-SO_3_H IL (10 mol%)	EtOH	Reflux	00:10	96	This study

^a^Reaction mixture: 4-nitrobenzaldehyde (1.0 mmol), dimedone (2.0 mmol), and ammonium acetate (1.0 mmol).

^b^Reaction mixture: 2-hydroxybenzaldehyde (1.0 mmol), dimedone (1.0 mmol), ethyl acetoacetate (1.0 mmol), and ammonium acetate (1.0 mmol).

^c^Isolated yields.

## Experimental

### Materials and instruments

All chemicals, solvents and substrates, involving iron(II) chloride tetrahydrate, iron(III) chloride hexahydrate, tetraethyl orthosilicate, 3-(triethoxysilyl)propan-1-amine, 2,4,6-trichloro-1,3,5-triazine, 1*H*-imidazole, 1,4-butane sultone, N(C_2_H_5_)_3_, NH_3_.H_2_O, EtOH, MeOH, toluene, THF, MeCN, EtOAc, *n*-hexane, ammonium acetate, aromatic aldehydes, dimedone, and ethyl acetoacetate were purchased from Sigma-Aldrich and Merck and used without further purification. All organic processes were checked and visualized by TLC with commercial aluminum-backed plates of silica gel 60 F254 by UV light, respectively. Melting points were determined in open capillaries using an Electrothermal 9100. ^1^H NMR and ^13^C NMR spectra were obtained using a Bruker DRX-400 spectrometer at 400 and 100 MHz respectively. Importantly, all reported products are known and some have been identified by FTIR and NMR analysis (Figs. S11–S40).

The n-Fe_3_O_4_@SiO_2_-TA-SO_3_H IL characterization was accomplished by using different techniques involving, Fourier transform infrared spectroscopy (FTIR), Scanning electron microscopy (SEM)/ Energy-dispersive X-ray analysis (EDX), Transmission Electron Microscopy (TEM), Vibrating sample magnetometry (VSM), Brunauer–Emmett–Teller (BET), X-ray diffraction (XRD), Thermogravimetric analysis (TGA) measurements. FTIR spectra were recorded by employing PERKIN-ELMER-Spectrum 65 instrument. SEM/EDX images were obtained by using a Tescan instrument, using Au-coated samples (20 kV). The n-Fe_3_O_4_@SiO_2_-TA-SO_3_H IL morphologies explored via TEM analysis by employing Philips CM30300Kv field emission transmission electron microscope. The magnetic properties of n-Fe_3_O_4_@SiO_2_-TA-SO_3_H IL and n-Fe_3_O_4_ were characterized employing VSM (Lakeshore7407). TGA was carried out using a METTLER TOLEDO apparatus over the range of 25–700 °C under nitrogen atmosphere (rating, 10 C min^-1^). XRD patterns were provided employing a Siemens, D5000.CuKα radiation by the sealed tube. The BET analyses were performed using BELSORP Mini II instrument (degassing of samples at 423 K for 2.5 h).

### Catalyst preparation

#### *Synthesis of n-Fe*_*3*_*O*_*4*_* (a)*

N-Fe_3_O_4_ were synthesized via an improved chemical co-precipitation fashion^45^. At first, FeCl_2_.4H_2_O (2.982 g, 10.0 mmol) and FeCl_3_.6H_2_O (8.108 g, 20.0 mmol) were initially dissolved in deionized water (75 mL), mixed homogeneously under argon atmosphere and reflux conditions for 3 h. After that, an aqueous ammonia solution (15 mL, 28%) was injected slowly into the above mixture with vigorous stirring. The mixture was stirred magnetically under reflux for 2 h and then allowed being cooled down to ambient temperature. The attained black n-Fe_3_O_4_ were washed successively with EtOH-water 3 times and separated using an external magnetic field. Afterwards, n-Fe_3_O_4_ dried at 50 °C for 12 h.

#### *Synthesis of n-Fe*_*3*_*O*_*4*_*@SiO*_*2*_* (b)*

The n-Fe_3_O_4_@SiO_2_ preparation was performed according to a previously reported fashion with slight modification^46^. Typically, n-Fe_3_O_4_ (1 g) well-dispersed in EtOH:deionized (40 mL, 4:1) and NH_3_.H_2_O (6 mL, 28%) and dispersed for 0.5 h. Notably, the pH of mixture was kept at *ca* 11. Further, tetraethyl orthosilicate (2.0 g, 9.6 mmol) was added into above-mixture slowly and stirred continuously at r.t. for 13 h under air. Finally, the n-Fe_3_O_4_@SiO_2_ was collected employing an external magnetic field. To purify and remove the unreacted substrates, n-Fe_3_O_4_@SiO_2_ was washed two times using EtOH-water and dried at 75 °C for 6.5 h.

#### *Synthesis of NH*_*2*_*-functionalized n-Fe*_*3*_*O*_*4*_*@SiO*_*2*_* (c)*

To prepare n-Fe_3_O_4_@SiO_2_-NH_2_, 3-(triethoxysilyl)propan-1-amine (0.8 g, 3.61 mmol) and n-Fe_3_O_4_@SiO_2_ (1.0 g) were mixed in dry toluene (50 mL) and well-dispersed under ultrasonic irradiation. After half an hours, the mentioned mixture was heated under reflux conditions and argon atmosphere for 24 h. At the end of the process, corresponding precipitate was separated employing a magnet, washed for 3 times with toluene/methanol and dried at 45 °C for 3 h.

#### *Synthesis of Synthesis of n-Fe*_*3*_*O*_*4*_*@SiO*_*2*_*-TA (d)*

For the preparation of n-Fe_3_O_4_@SiO_2_-TA, 2,4,6-Trichloro-1,3,5-triazine (0.85 g, 4.6 mmol) was dissolved in dry-THF (20 mL) in the ice-bath under argon atmosphere. Later, 1 g of well-dispersed n-Fe_3_O_4_@SiO_2_-NH_2_ in 10 mL THF was added to triazine solution and stirred vigorously overnight. At the final step, the n-Fe_3_O_4_@SiO_2_-TA was magnetically separated, washed for 3 times with acetone/THF, and dried for 4 h at 40 °C.

#### Synthesis of 3-(n-butanesulfonate)-imidazole (e)

3-(*N*-butanesulfonate)-imidazole was prepared according to the reported literatures^47–49^. In this step, 1*H*-imidazole (0.916 g, 13.45 mmol) was completely dissolved in dry-MeCN (10 mL) at 50 °C and 1,4-butane sultone (1.666 g, 12.23 mmol) was added dropwisely into the imidazole solution under dark conditions and argon atmosphere for the duration 12 h. Afterward, 3-(*n*-butanesulfonate)-imidazole obtained when the solvent was removed and washed with and dried for 3.5 h in 50 °C.

#### *Synthesis of n-Fe*_*3*_*O*_*4*_*@SiO*_*2*_*-TA-SO*_*3*_*H IL (f)*

**Figure 5:**
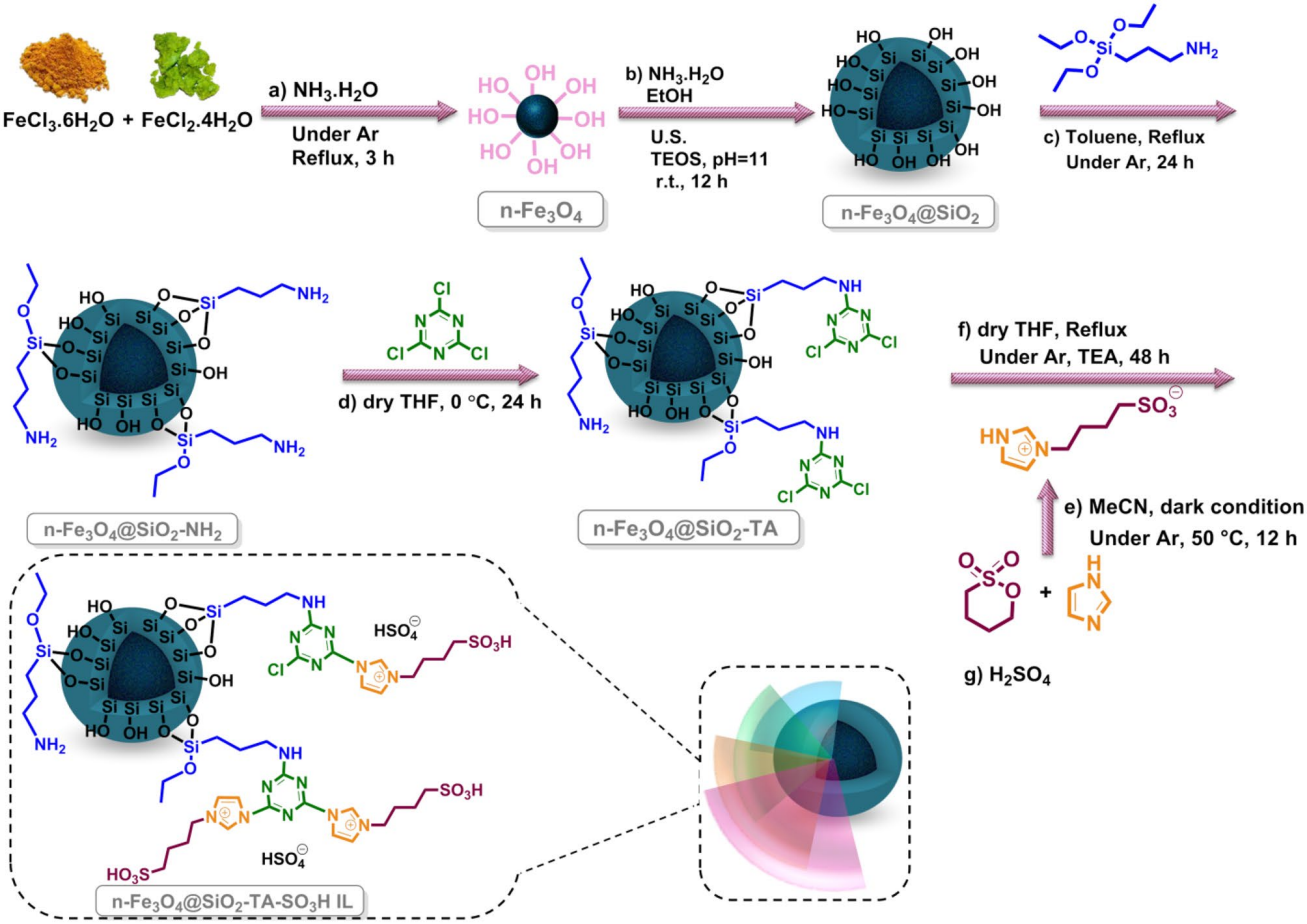
The possible formation process of n-Fe_3_O_4_@SiO_2_-TA-SO_3_H IL.

3-(*N*-butanesulfonate)-imidazole (0.86 g) was completely dissolved in dry-THF (20 mL) and refluxed under Ar atmosphere for 15 min. Next, n-Fe_3_O_4_@SiO_2_-TA (1.0 g) that dissolved in dry-THF (20 mL), added into 3-(*n*-butanesulfonate)-imidazole solution and the as-prepared mixture was stirred overnight. A solution of 3-(*n*-butanesulfonate)-imidazole (0.86 g) in dry-THF (20 mL) and triethylamine (3 drops) was added into above-mixture. Subsequently, the above mixture was refluxed under Ar atmosphere for 24 h. Subsequently, n-Fe_3_O_4_@SiO_2_-TA-SO_3_^–^ IL was collected magnetically, washed with methanol/THF and dried at r.t. overnight. Finally, H_2_SO_4_ (3 mL) was slowly added. Afterward n-Fe_3_O_4_@SiO_2_-TA-SO_3_H IL was collected magnetically and dried for 4 h in 60 °C (Fig. 5).

### Catalytic activity

#### General procedure for symmetric HnR

A mixture of an aldehyde (1.0 mmol), ammonium acetate (1.0 mmol), dimedone or ethyl acetoacetate (2.0 mmol), and n-Fe_3_O_4_@SiO_2_-TA-SO_3_H IL (10 mol%) were added into a round-bottomed flask in H_2_O (5 mL) and stirred under reflux conditions. The progress of the reaction was monitored by TLC (EtOAc/*n*-hexane, (2:3)). After completion of the reaction, the n-Fe_3_O_4_@SiO_2_-TA-SO_3_H IL was easily isolated by an external magnetic. The pure product was attained in high yield after recrystallization from EtOH/H_2_O.

#### General procedure for asymmetric HnR

A mixture of ammonium acetate (1.0 mmol), aromatic aldehyde (1.0 mmol), dimedone (1.0 mmol), ethyl acetoacetate (1.0 mmol), and n-Fe_3_O_4_@SiO_2_-TA-SO_3_H IL (10 mol%) were added into a round-bottomed flask in EtOH (5 mL) and stirred under reflux conditions. Notably, reaction progress was checked by TLC (EtOAc/*n*-hexane, (2:3)). After completion of the reaction, the n-Fe_3_O_4_@SiO_2_-TA-SO_3_H IL was easily isolated by an external magnetic. The pure product was attained in high yield after recrystallization from EtOH/H_2_O.

## Conclusion

Combining the advantages of n-Fe_3_O_4_ and ILs, n-Fe_3_O_4_@SiO_2_-TA-SO_3_H IL was designed and synthesized via step by step functionalization of n-Fe_3_O_4_@SiO_2_ using TCT followed by covalent reaction with imidazolium-ILs. The as-prepared catalyst efficaciously used for catalyzing the synthesis of 1,8-DOXDHA, 1,4-DHP, and PHQ derivatives through HnR. It can be stated that the ILs-functionalization of n-Fe_3_O_4_@SiO_2_-TA can intensely increase the greater catalytic activity of n-Fe_3_O_4_@SiO_2_-TA-SO_3_H IL. Moreover, the recyclability examination also established that n-Fe_3_O_4_@SiO_2_-TA-SO_3_H IL was extraordinary reusable and could be recovered for up to eight reaction runs.

## Supplementary Information

Supplementary Information.

## Data Availability

All data generated or analyzed during this study are included in this published article (and its Supplementary Information files).
