# Supplementary material for: Ionic liquid-loaded triazine-based magnetic nanoparticles for promoting multicomponent reaction
Source: Sci Rep. 2022 Dec 23;12:22261. doi: 10.1038/s41598-022-26235-6 (PMC9789048; doi:10.1038/s41598-022-26235-6)
Supplement: Supplementary file 1 — Supplementary Information. [file 41598_2022_26235_MOESM1_ESM.pdf]

Supporting information

Ionic liquid-loaded triazine-based magnetic nanoparticles for promoting multicomponent reaction

Kosar Kafshdarzadeh, Masoume Malmir, Zahra Amiri, Majid M. Heravi*

*Department of Organic Chemistry, Faculty of Chemistry, Alzahra University, Vanak, Tehran, Iran

Tel.: +98 9121329147; fax: +98 2188041344; E-mail: mmheravi@alzahra.ac.ir

Table S1. Optimization of reaction conditions in the symmetrical HnR[a]

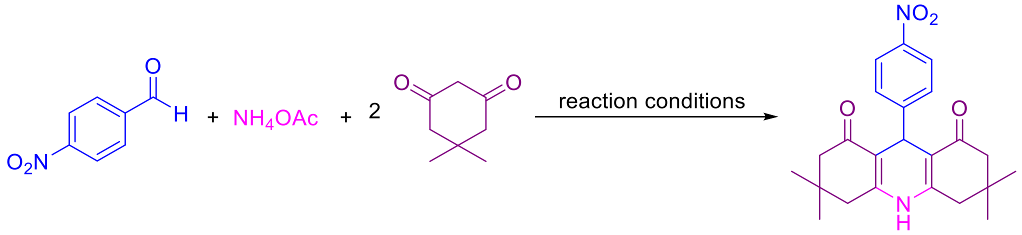

1b 2 3a 4b

Entry	Solvent	Temperature (°C)	Catalyst	Amount of catalyst (mol%)	Time (h:min)	Yield[b] (%)
1	H_2O	r.t.	n-Fe_3O_4@SiO_2-TA-SO_3H IL	10 mol%	01:23	91
2	H_2O	50	n-Fe_3O_4@SiO_2-TA-SO_3H IL	10 mol%	00:22	93
**3**	**H_2O**	**Reflux**	**n-Fe_3O_4@SiO_2-TA-SO_3H IL**	**10 mol%**	**00:08**	**97**
4	EtOH	Reflux	n-Fe_3O_4@SiO_2-TA-SO_3H IL	10 mol%	00:07	89
5	H_2O/EtOH (2:1)	Reflux	n-Fe_3O_4@SiO_2-TA-SO_3H IL	10 mol%	00:10	83
6	DMF	Reflux	n-Fe_3O_4@SiO_2-TA-SO_3H IL	10 mol%	00:08	Trace
7	THF	Reflux	n-Fe_3O_4@SiO_2-TA-SO_3H IL	10 mol%	00:08	55
8	MeCN	Reflux	n-Fe_3O_4@SiO_2-TA-SO_3H IL	10 mol%	00:08	65
9	$CHCl_3$	Reflux	n-Fe_3O_4@SiO_2-TA-SO_3H IL	10 mol%	00:08	30
10	MeOH	Reflux	n-Fe_3O_4@SiO_2-TA-SO_3H IL	10 mol%	00:08	80
11	CH_2Cl_2	Reflux	n-Fe_3O_4@SiO_2-TA-SO_3H IL	10 mol%	00:08	40
12	Toluene	Reflux	n-Fe_3O_4@SiO_2-TA-SO_3H IL	10 mol%	00:08	55
13	H_2O	Reflux	n-Fe_3O_4@SiO_2-TA-SO_3H IL	5 mol%	00:08	82
14	H_2O	Reflux	n-Fe_3O_4@SiO_2-TA-SO_3H IL	20 mol%	00:08	70
15	H_2O	Reflux	-	-	00:08	Trace

[a] Reaction were run in solvent (5.0 mL), 4-nitrobenzaldehyde (1.0 mmol), ammonium acetate (1.0 mmol), dimedone (2.0 mmol)

[b] Isolated yield

Table S2. Optimization of reaction conditions in the asymmetrical HnR[a]

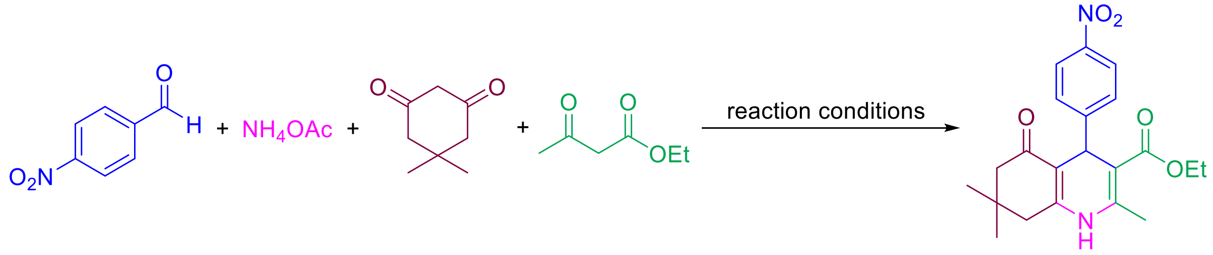

1b 2 3a 3b 6b

Entry	Solvent	Temperature (˚C)	Catalyst	Amount of catalyst (mol%)	Time (h:min)	Yield[b] (%)
1	H_2O	Reflux	n-Fe_3O_4@SiO_2-TA-SO_3H IL	10 mol%	01:15	90
2	H_2O/EtOH (2:1)	Reflux	n-Fe_3O_4@SiO_2-TA-SO_3H IL	10 mol%	01:50	80
**3**	**EtOH**	**Reflux**	**n-Fe_3O_4@SiO_2-TA-SO_3H IL**	**10 mol%**	**00:50**	**90**
4	MeCN	Reflux	n-Fe_3O_4@SiO_2-TA-SO_3H IL	10 mol%	00:50	50
5	MeOH	Reflux	n-Fe_3O_4@SiO_2-TA-SO_3H IL	10 mol%	00:50	68
6	EtOH	r.t.	n-Fe_3O_4@SiO_2-TA-SO_3H IL	10 mol%	00:50	65
7	EtOH	50	n-Fe_3O_4@SiO_2-TA-SO_3H IL	10 mol%	00:50	70
8	EtOH	Reflux	n-Fe_3O_4@SiO_2-TA-SO_3H IL	5 mol%	00:50	60
9	EtOH	Reflux	n-Fe_3O_4@SiO_2-TA-SO_3H IL	20 mol%	00:50	85
10	EtOH	Reflux	-	-	00:50	10

[a] Reaction were run in solvent (5.0 mL), 4-nitrobenzaldehyde (1.0 mmol), ammonium acetate (1.0 mmol), dimedone (1.0 mmol), ethyl acetoacetate (1.0 mmol)

[b] Isolated yield

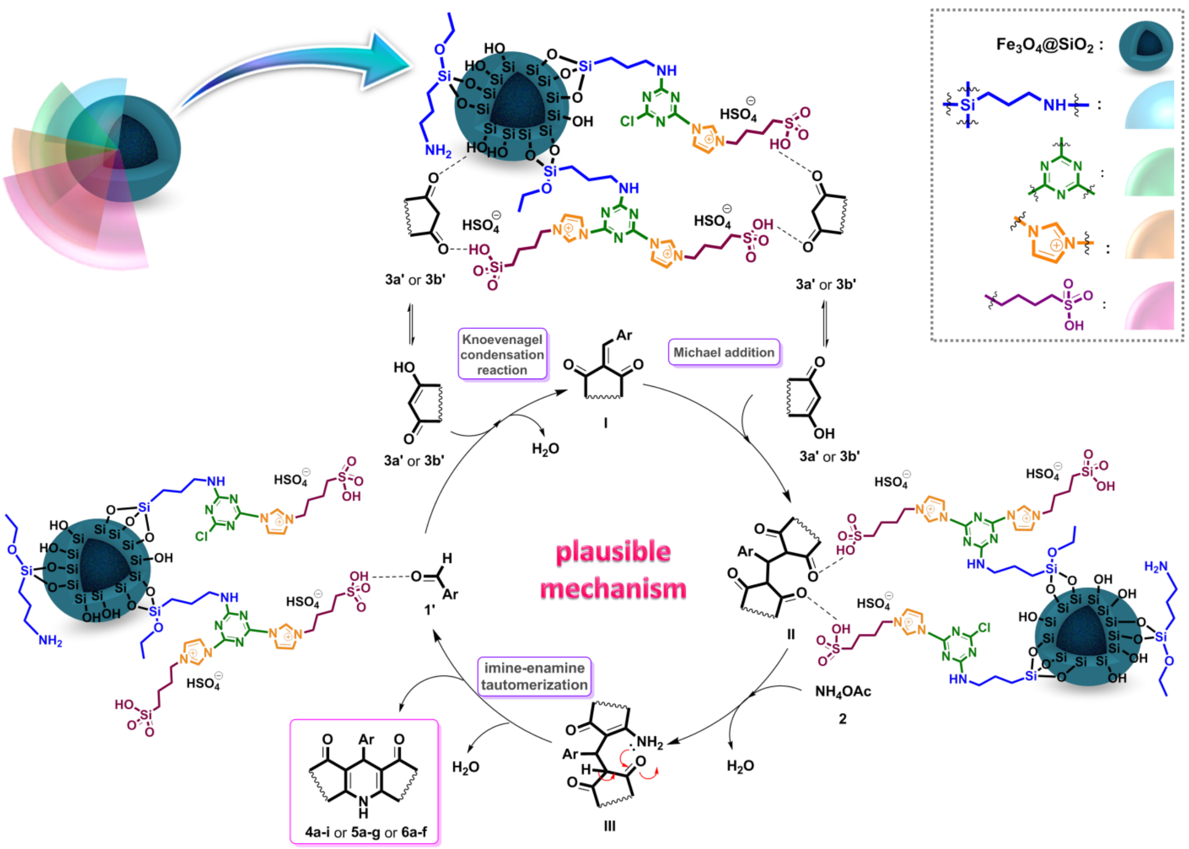

Figure S1. The plausible mechanism for the synthesis of 1,4-DHP (**5a-g**), 1,8-DOXDHA (**4a-i**) and PHQ derivatives (**6a-f**).

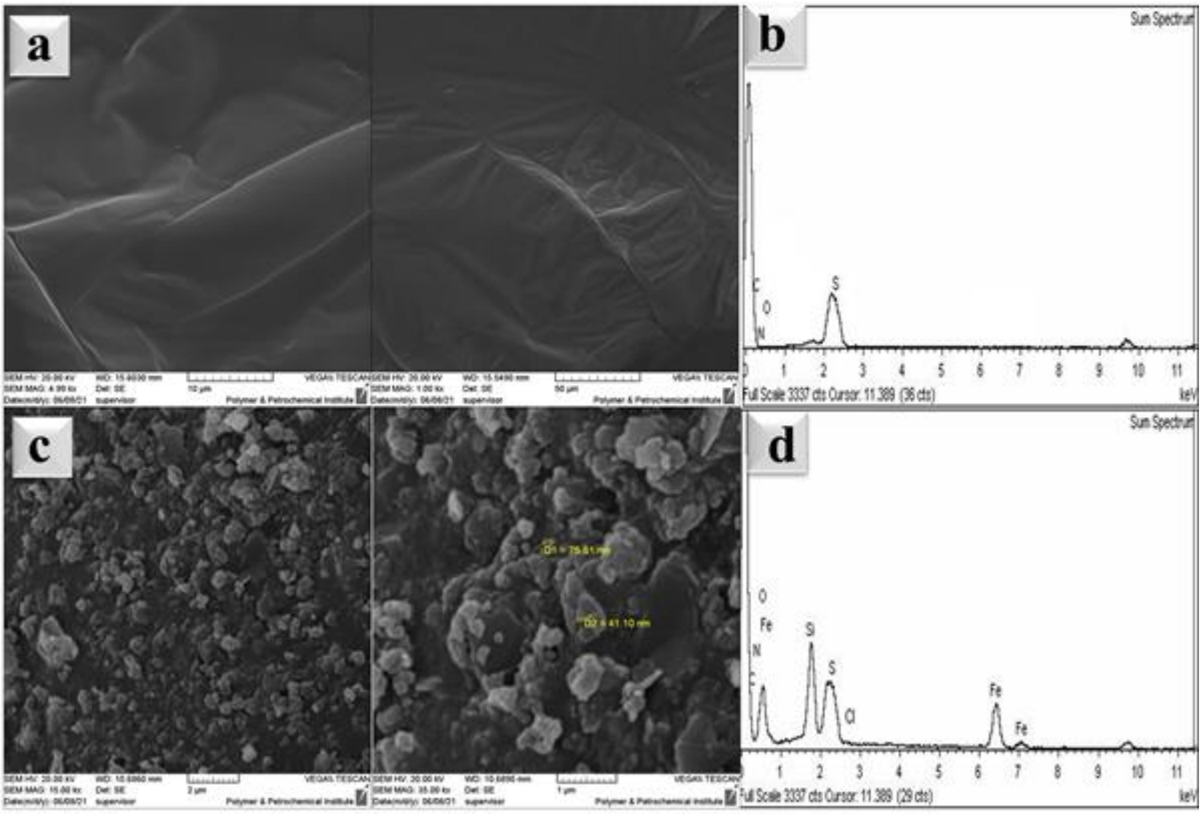

Figure S2. The SEM images and EDX analysis of ILs (a, b) and n-Fe_3O_4@SiO_2-TA-SO_3H IL(c, d).

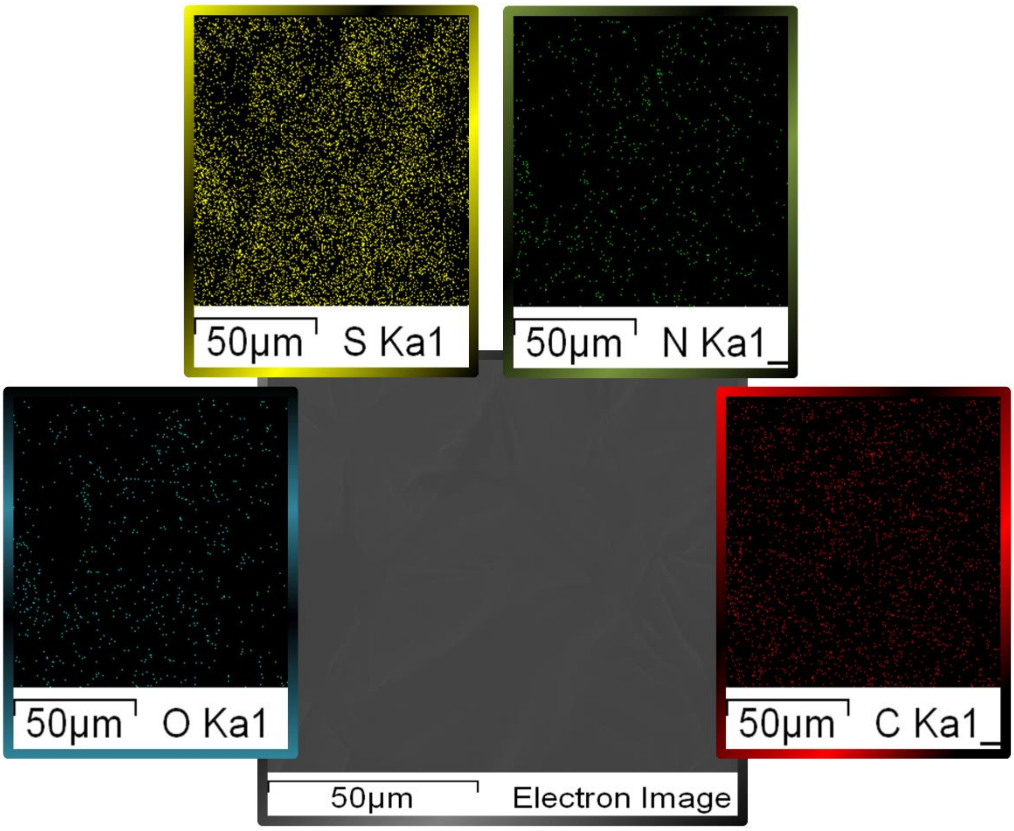

Figure S3. The elemental mapping analysis of ILs.

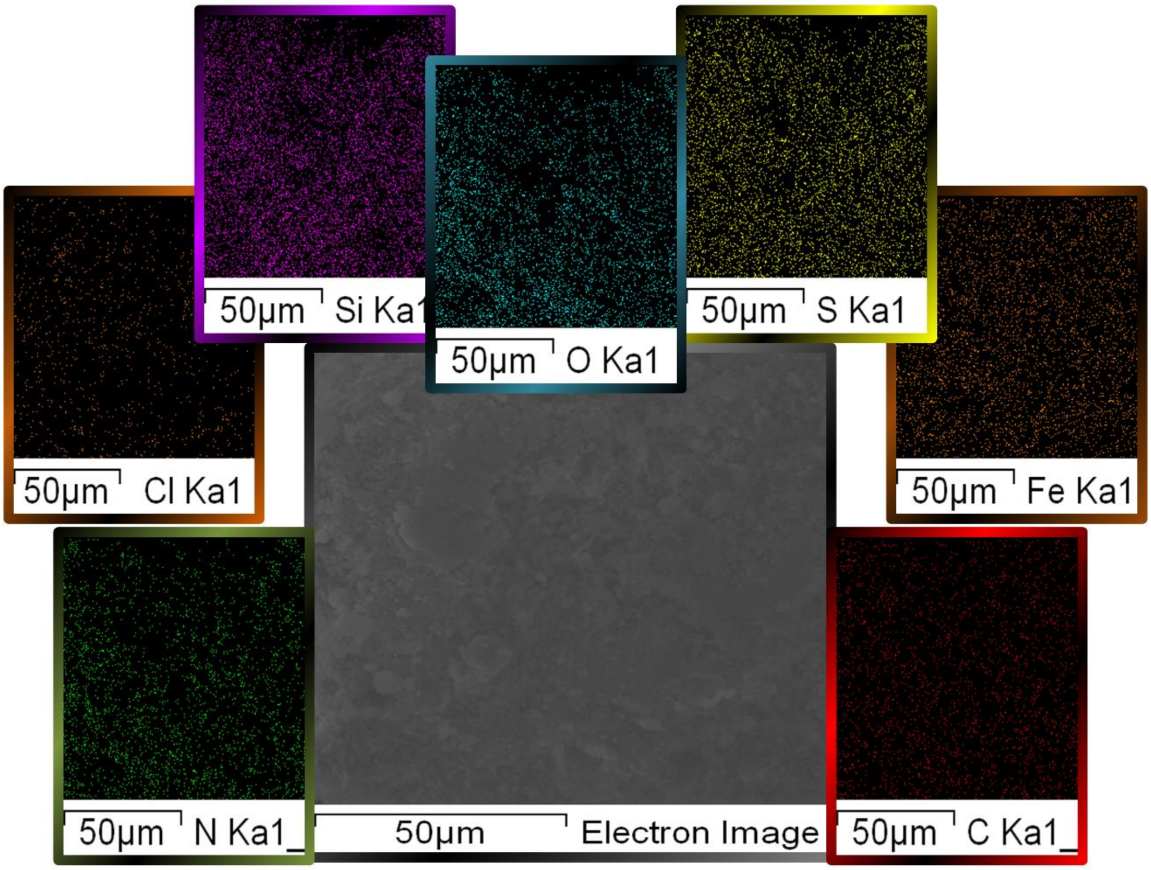

Figure S4. The elemental mapping analysis of n-Fe_3O_4@SiO_2-TA-SO_3H IL.

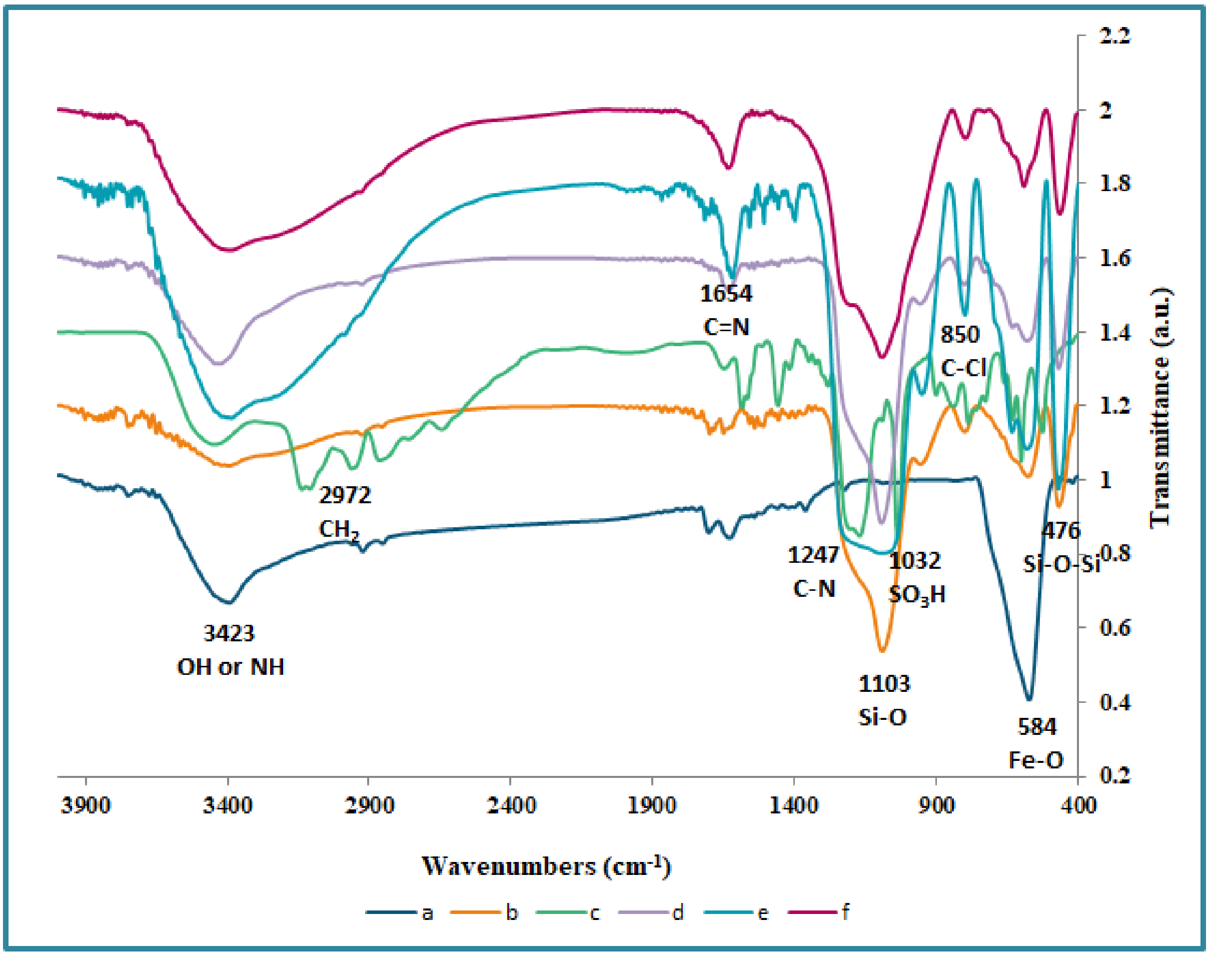

Figure S5. FT-IR spectrum of a) n-Fe_3O_4, b) n-Fe_3O_4@SiO_2, c) 3-(*n*-butanesulfonate)-imidazole, d) n-Fe_3O_4@SiO_2-NH_2, e) n-Fe_3O_4@SiO_2-TA, f) n-Fe_3O_4@SiO_2-TA-SO_3H IL.

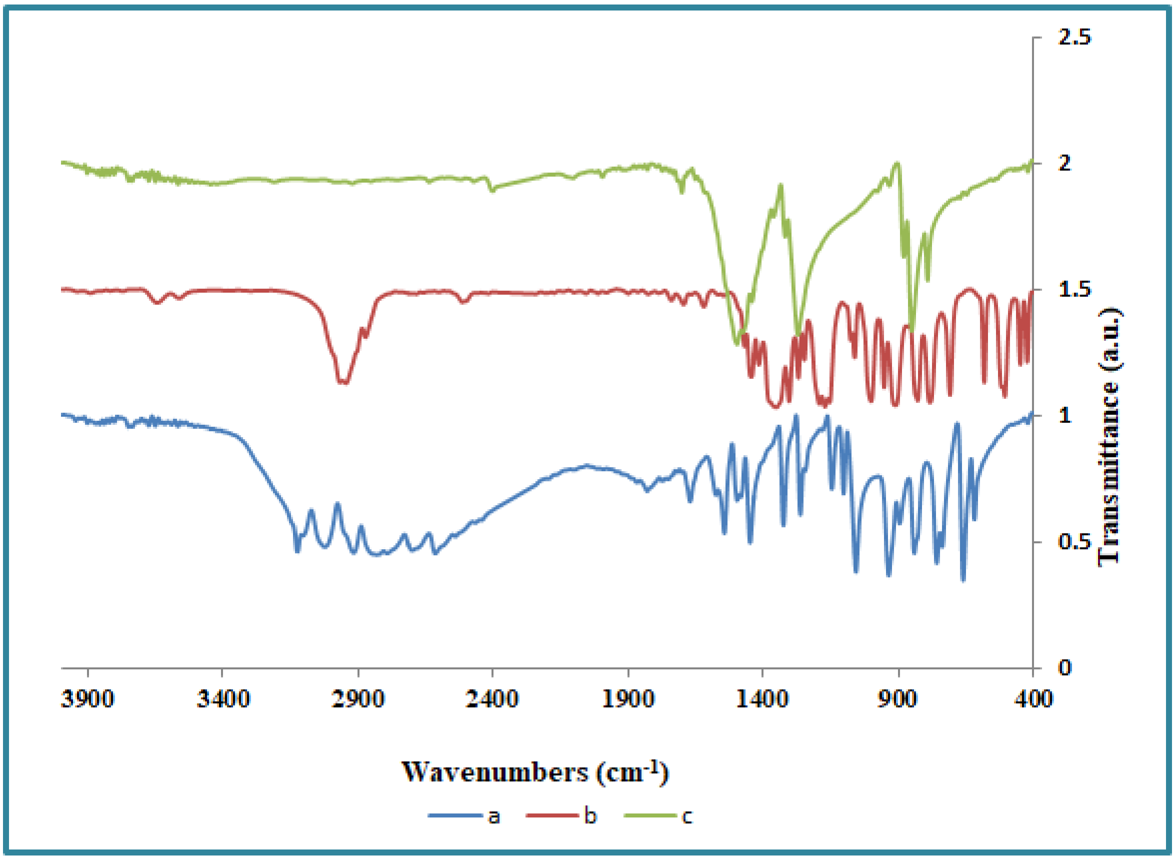

Figure S6. FT-IR spectra of a) 1*H*-imidazole, b) 1,4-butane sultone, c) 2,4,6-trichloro-1,3,5-triazine.

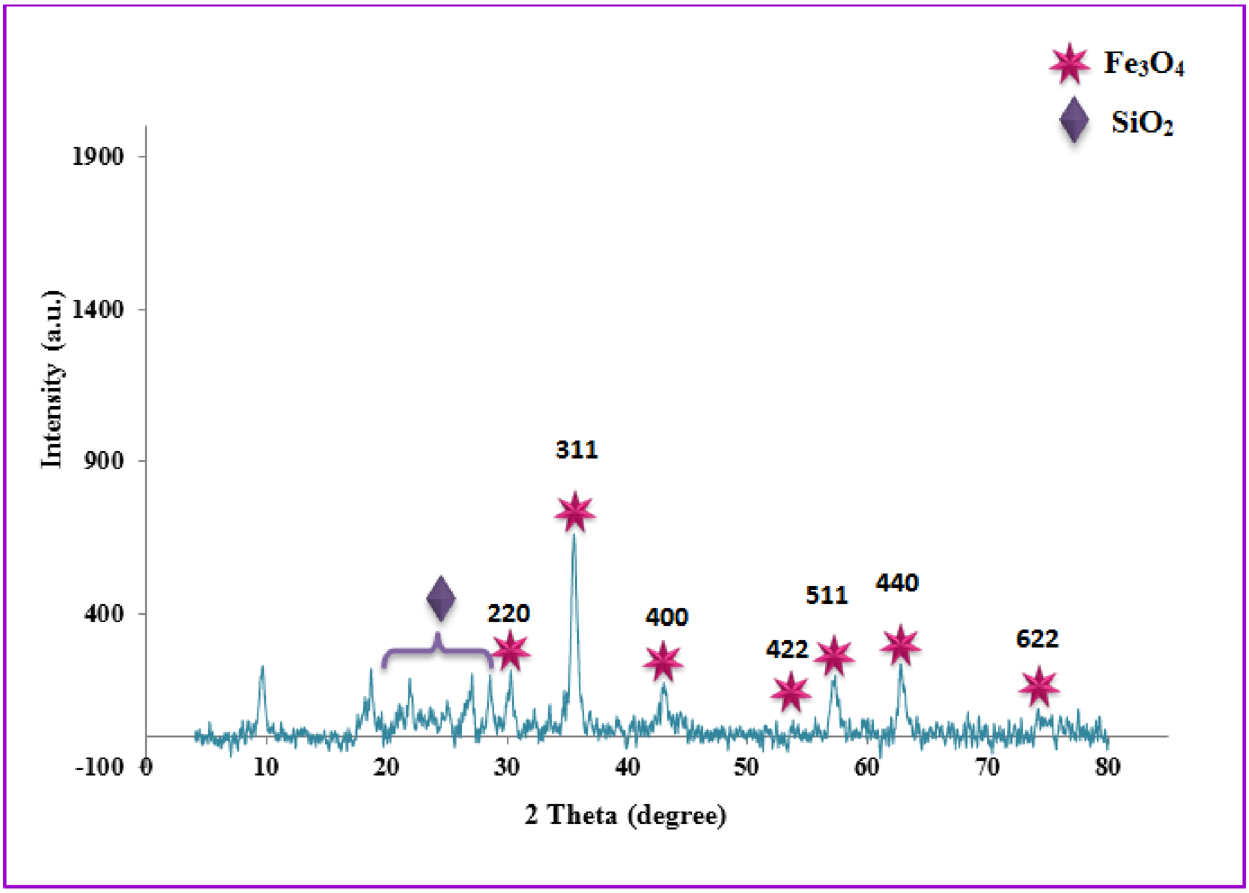

Figure S7. The XRD pattern of n-Fe_3O_4@SiO_2-TA-SO_3H IL.

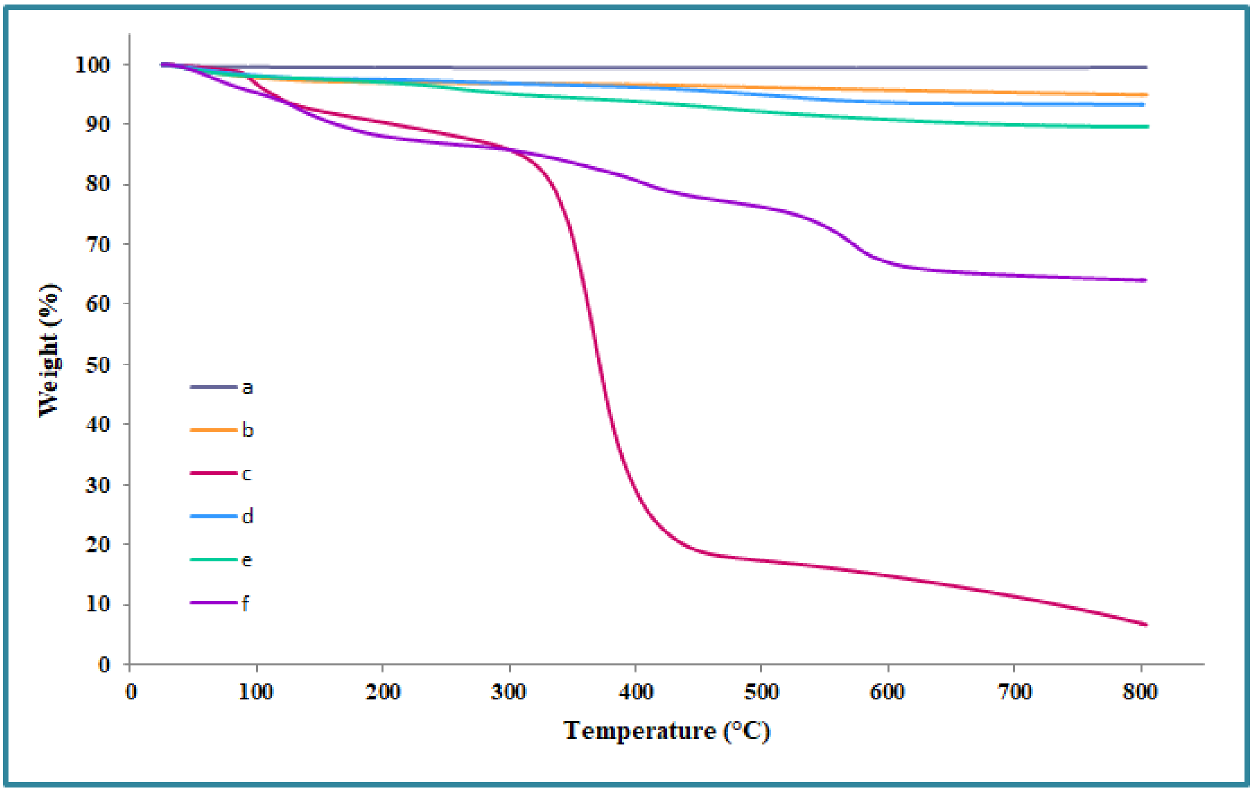

Figure S8. TG analyses of a) n-Fe_3O_4, b) n-Fe_3O_4@SiO_2, c) 3-(*n*-butanesulfonate)-imidazole, d) n-Fe_3O_4@SiO_2-NH_2, e) n-Fe_3O_4@SiO_2-TA, f) n-Fe_3O_4@SiO_2-TA-SO_3H IL.

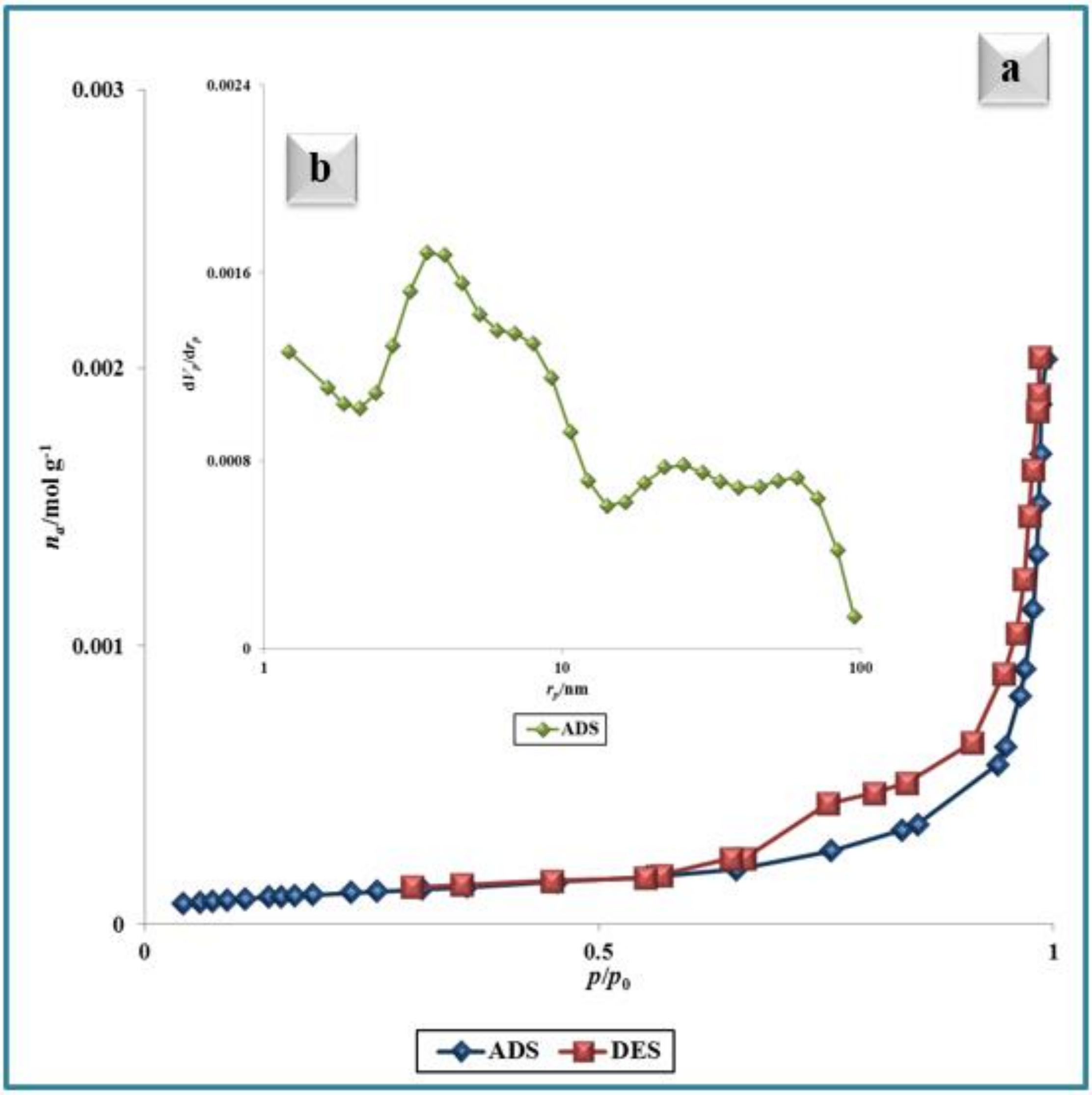

Figure S9. N_2-adsorption-desorption isotherms (a) and BJH-Plot (b) of n-Fe_3O_4@SiO_2-TA-SO_3H IL.

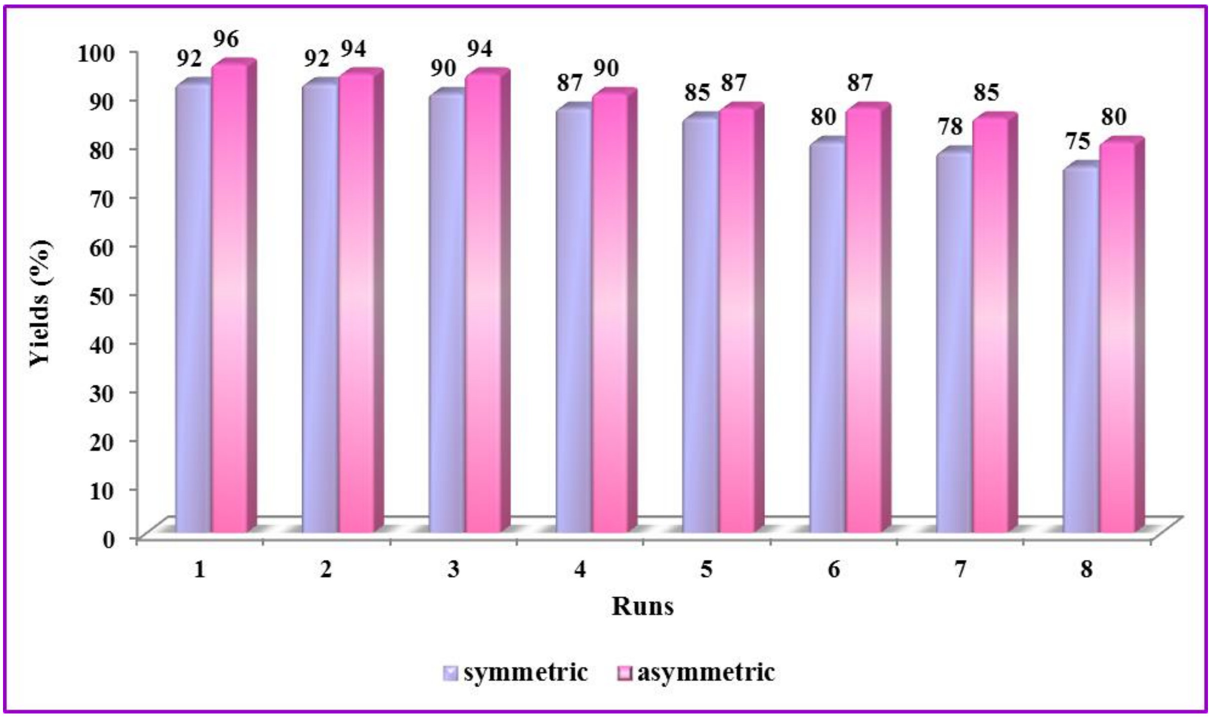

Figure S10. Recycling diagram of the n-Fe_3O_4@SiO_2-TA-SO_3H IL catalyst in the synthesis of **5e** and **6c**.

Copies of FTIR and ^{1}H-NMR for selected products

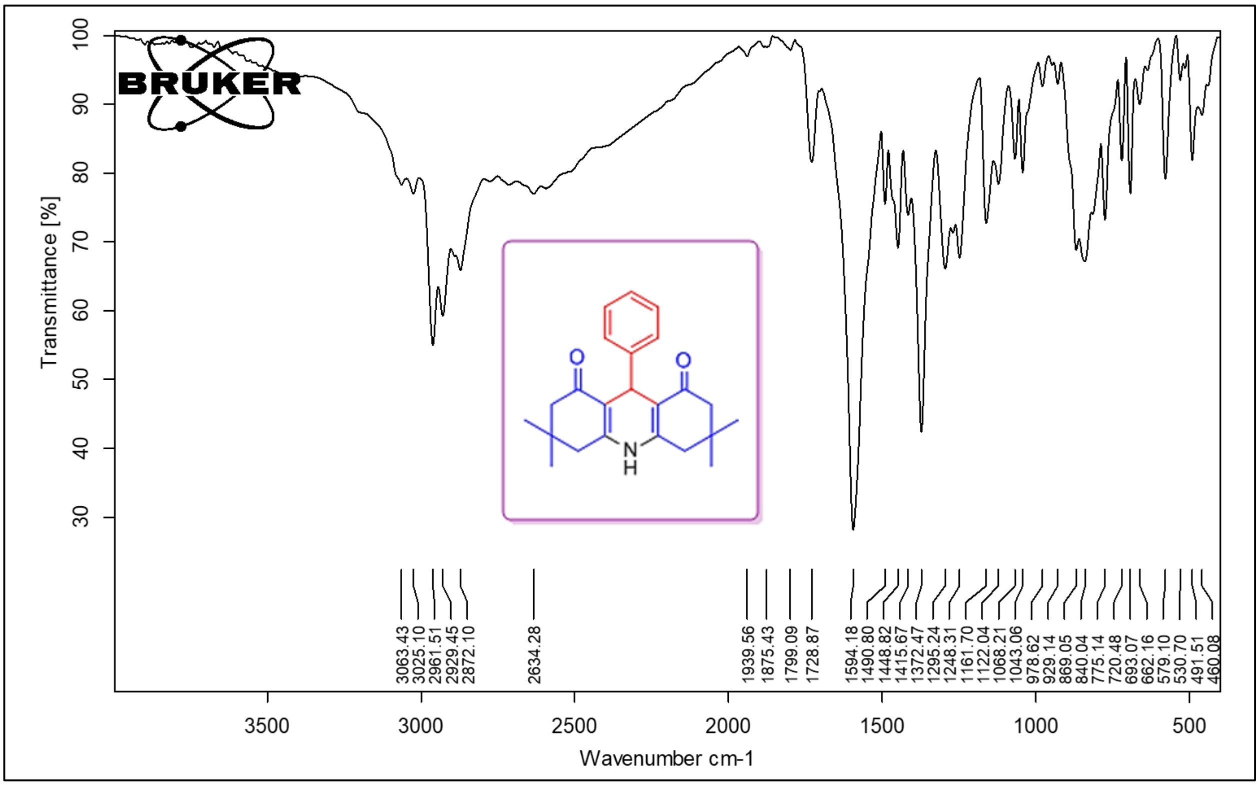

Figure S11. FTIR spectra of **(Table 1, 4a)**.

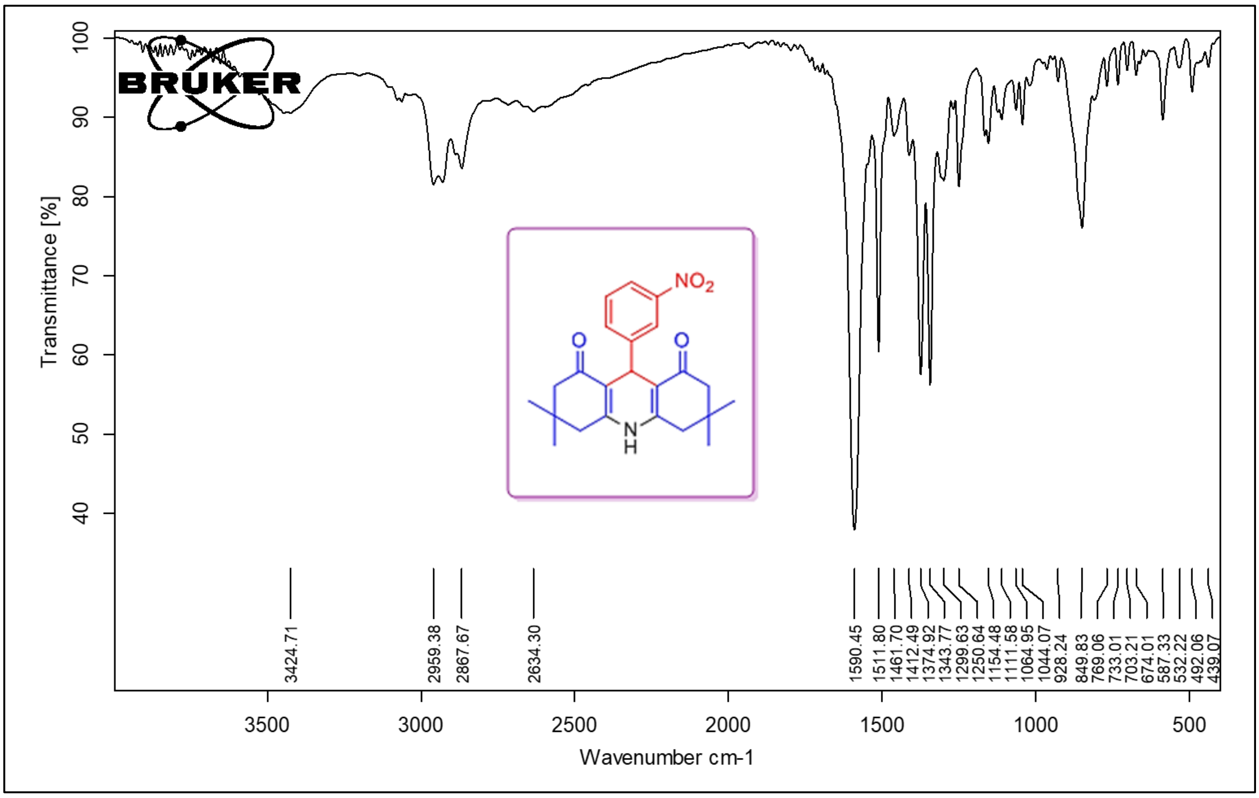

Figure S12. FTIR spectra of **(Table 1, 4b)**.

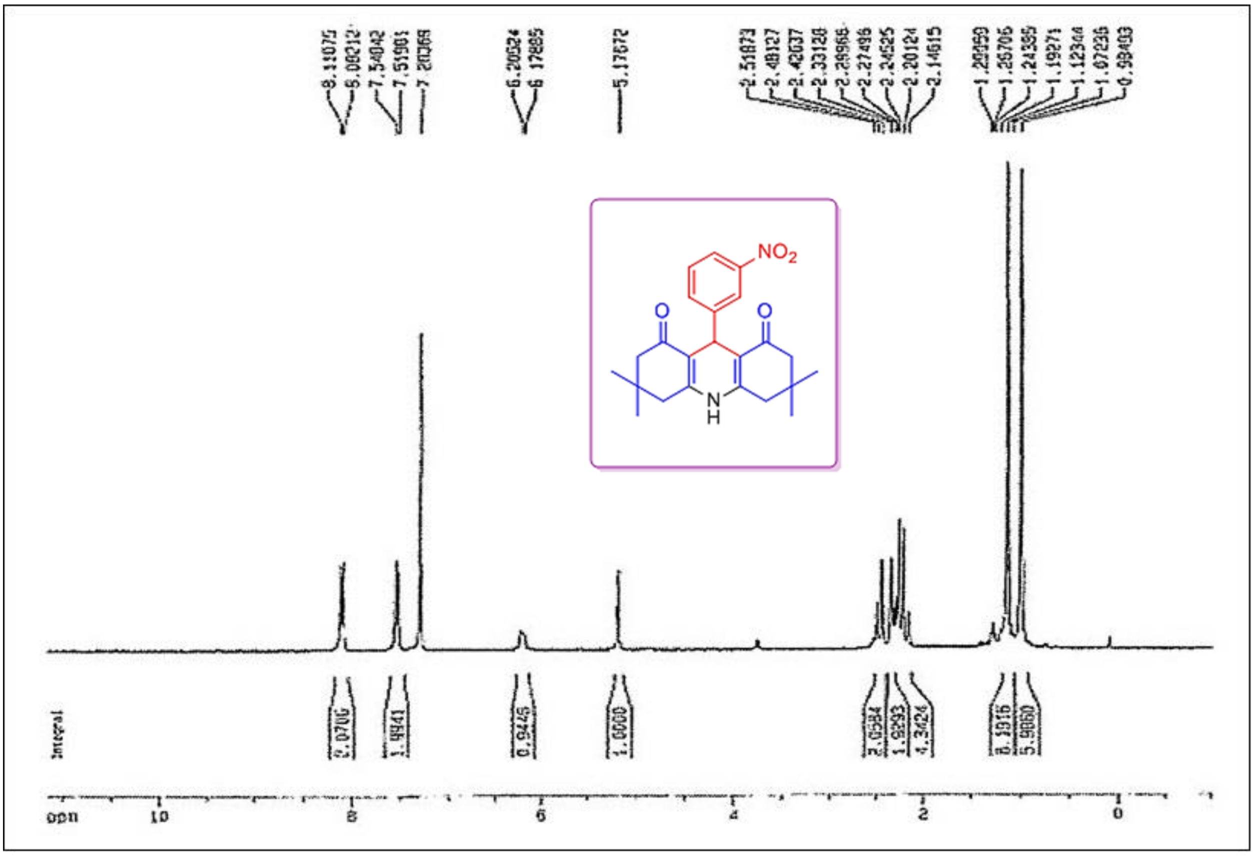

Figure S13. ^{1}H-NMR spectra of **(Table 1, 4b)**.

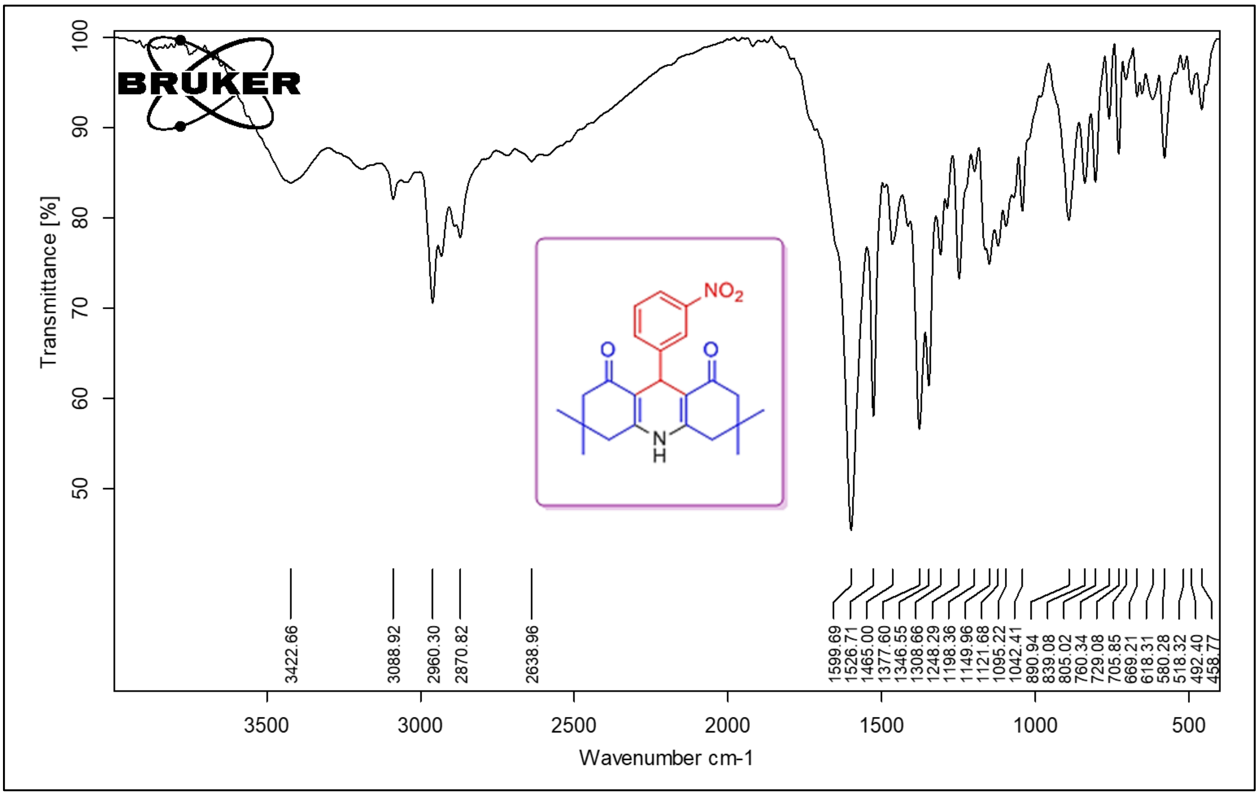

Figure S14. FTIR spectra of **(Table 1, 4c)**.

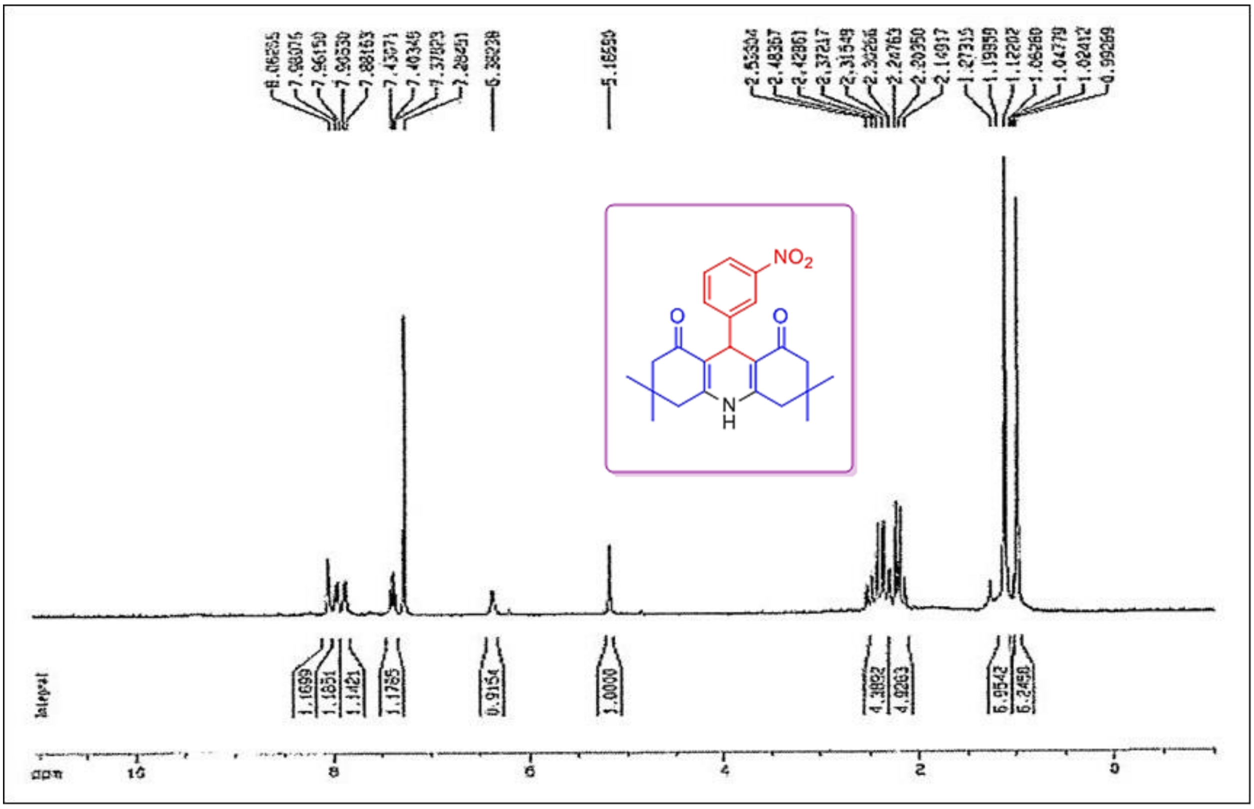

Figure S15. ^{1}H-NMR spectra of **(Table 1, 4c)**.

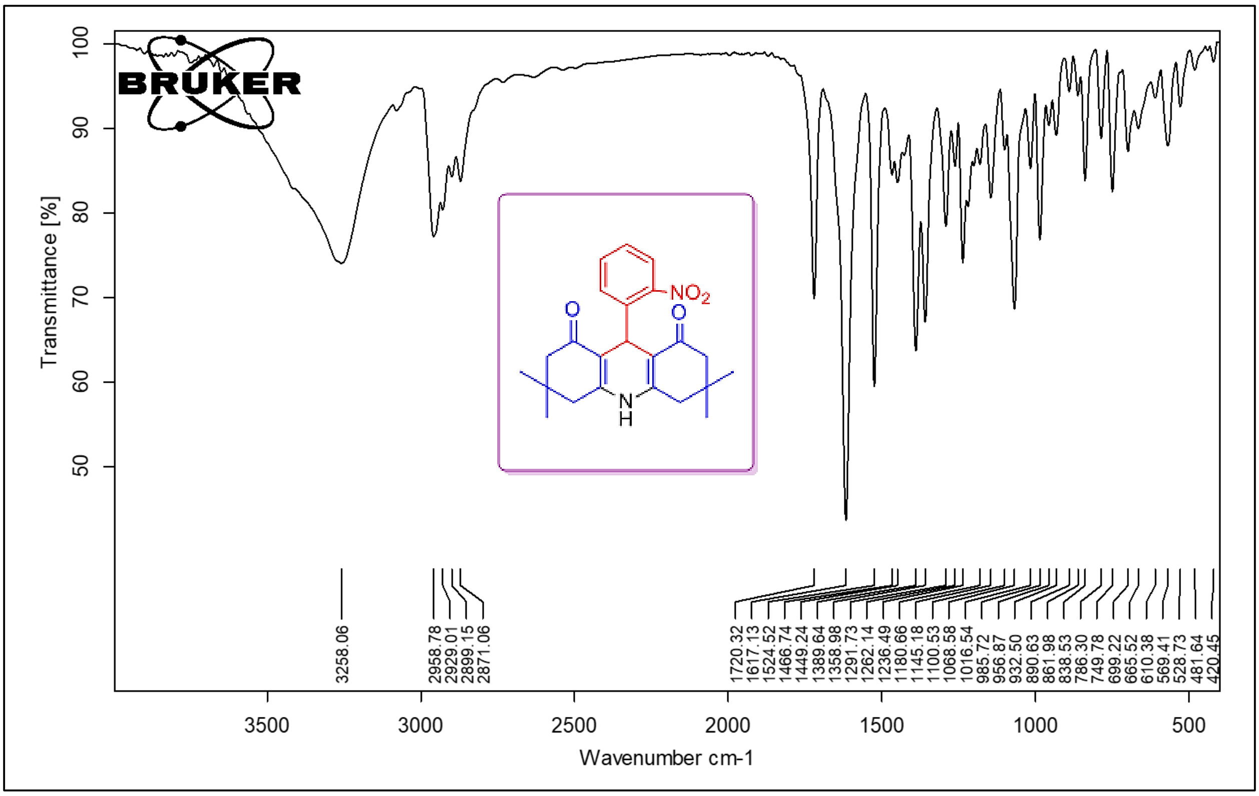

Figure S16. FTIR spectra of **(Table 1, 4d)**.

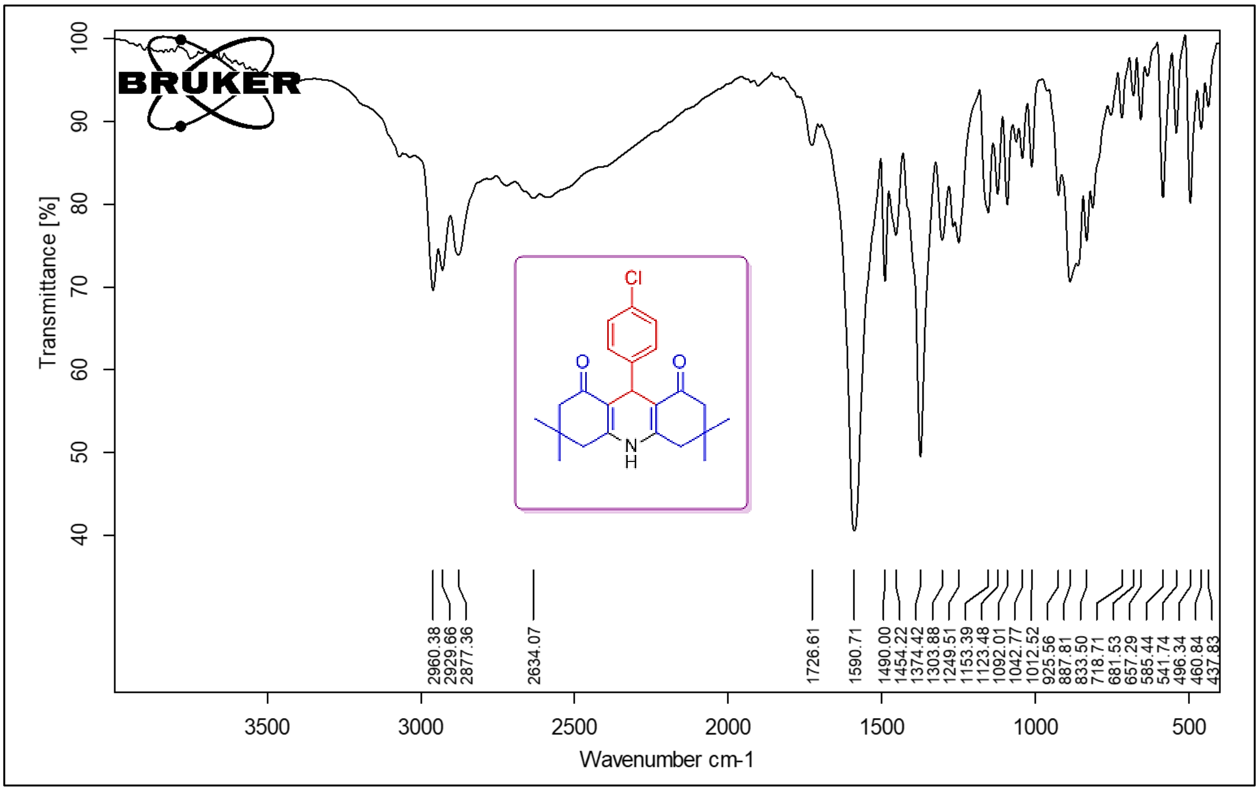

Figure S17. FTIR spectra of **(Table 1, 4e)**.

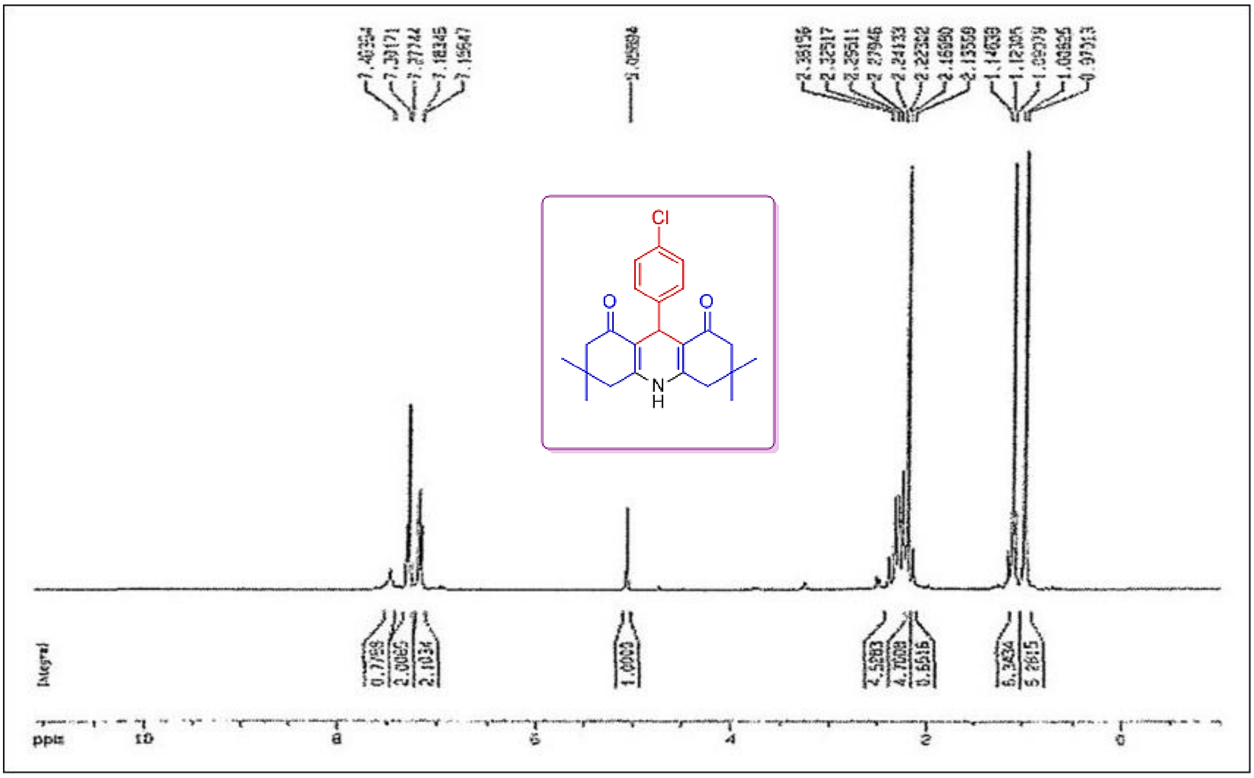

Figure S18. ^{1}H-NMR spectra of **(Table 1, 4e)**.

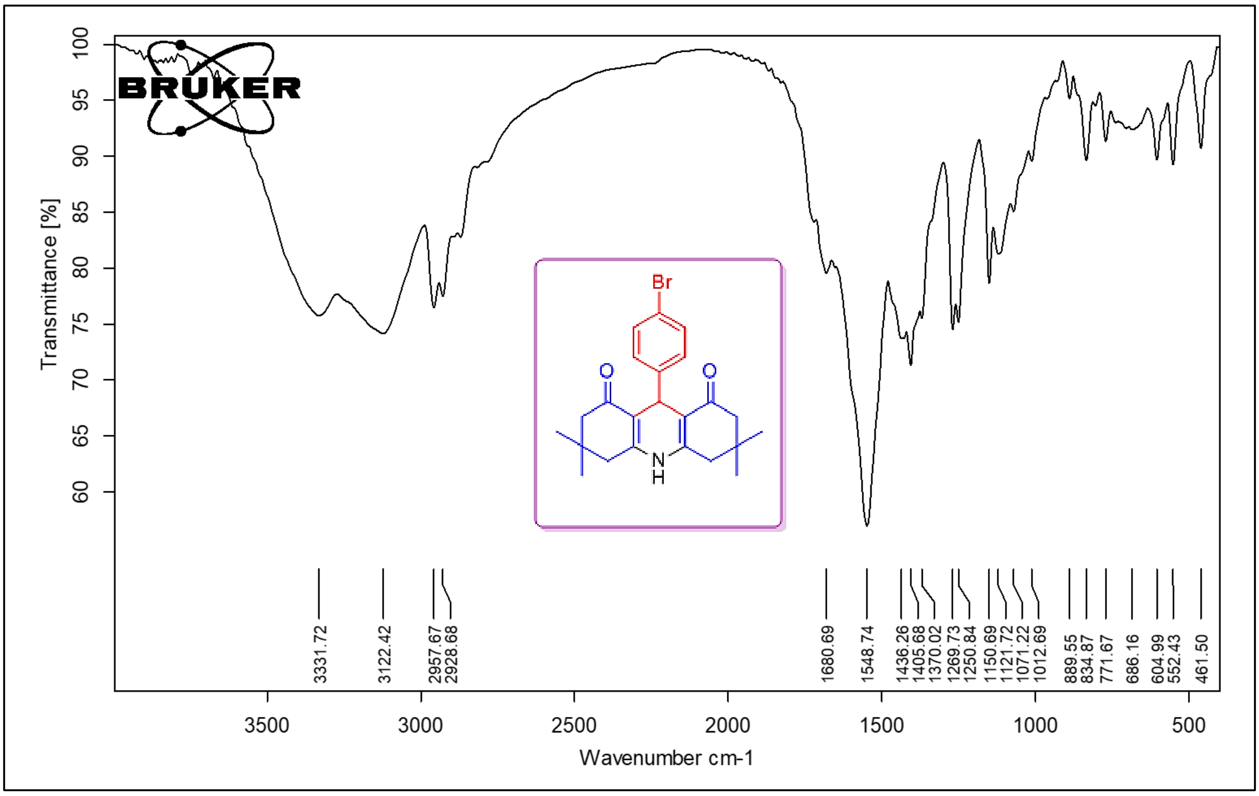

Figure S19. FTIR spectra of **(Table 1, 4f)**.

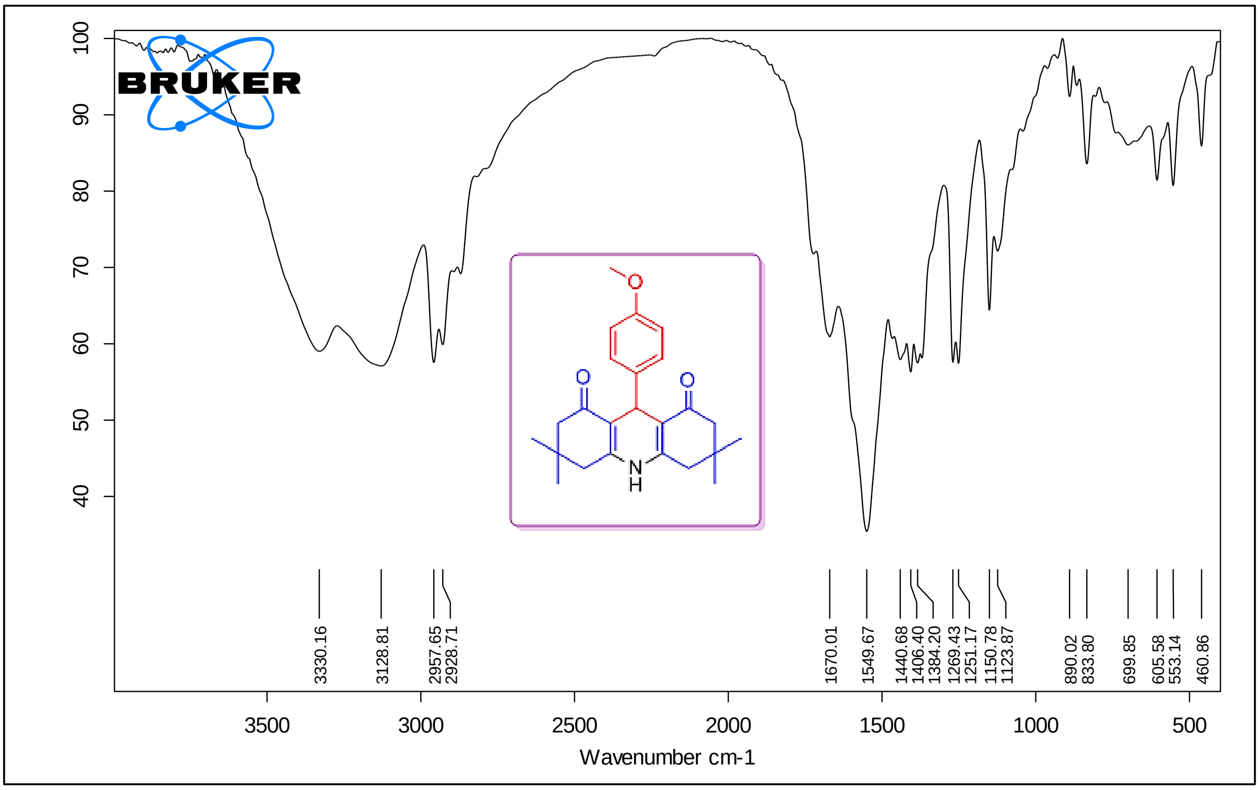

Figure. S20. FTIR spectra of **(Table 1, 4g)**.

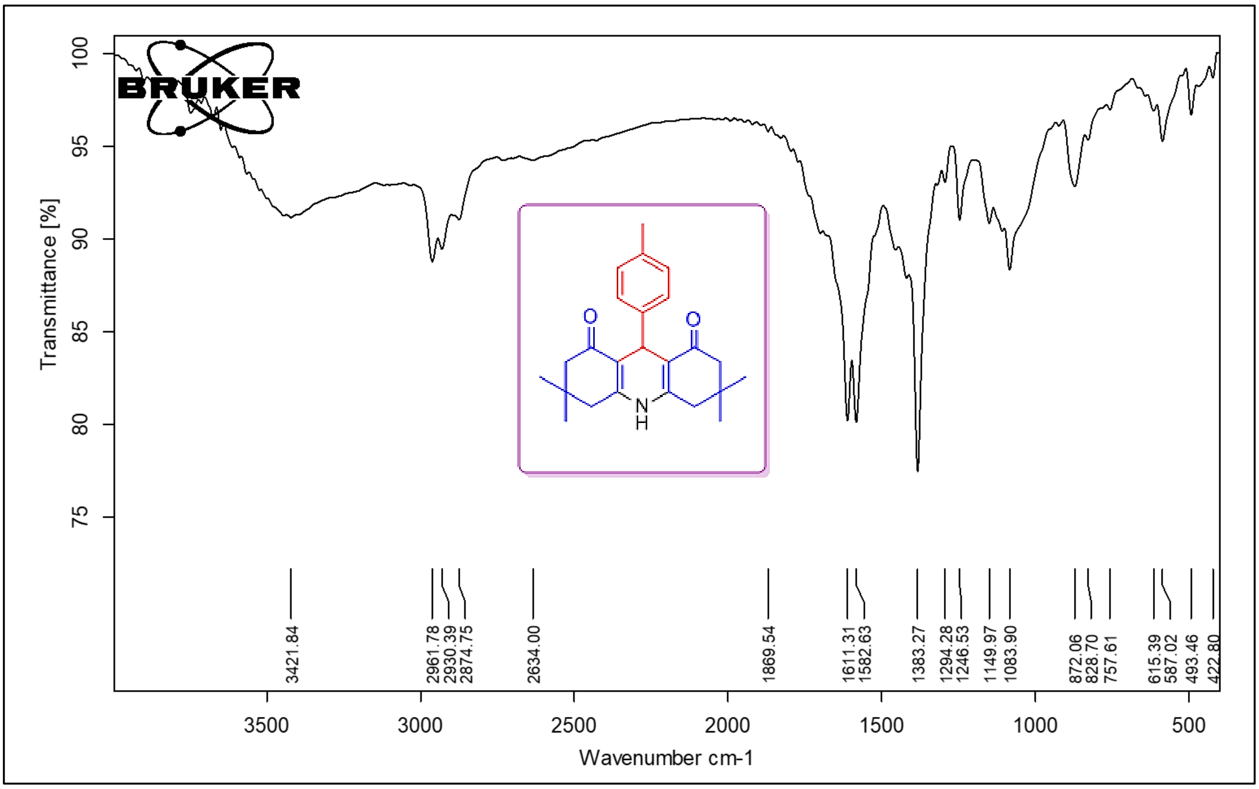

Figure S21. FTIR spectra of **(Table 1, 4h)**.

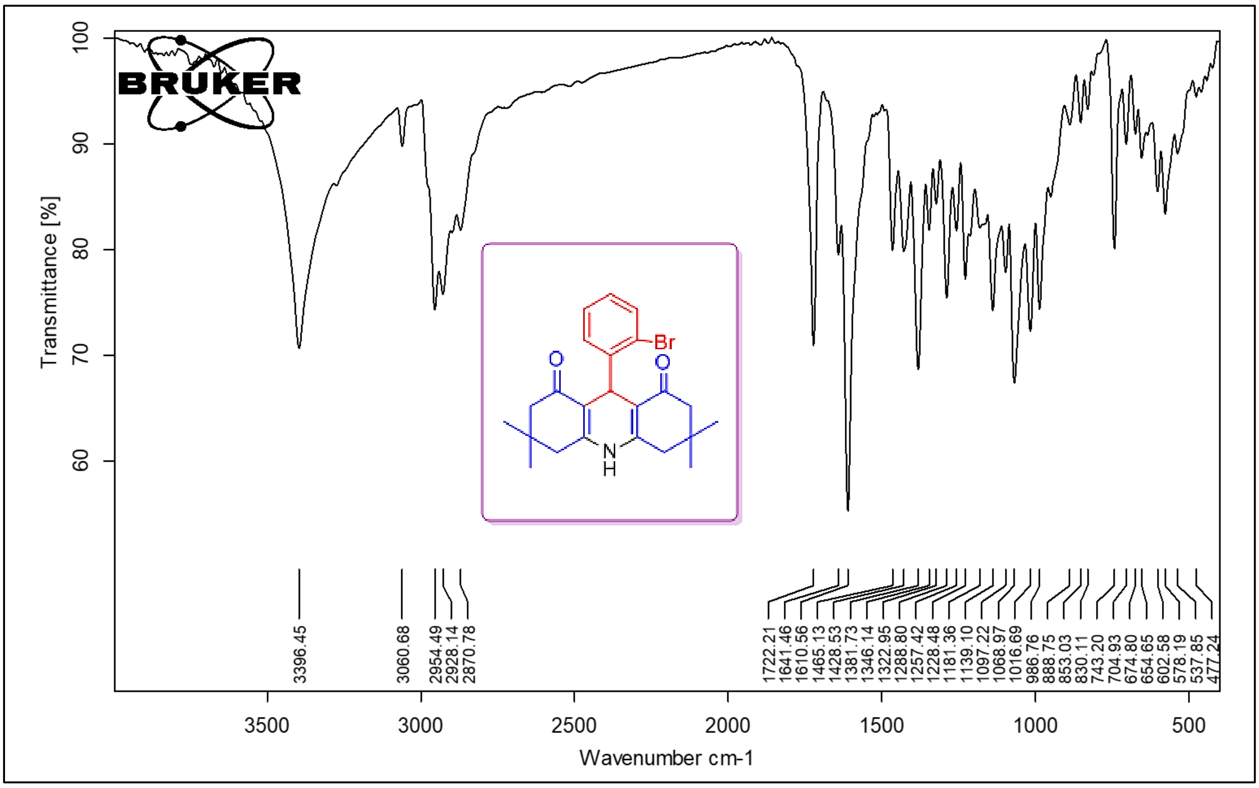

Figure S22. FTIR spectra of **(Table 1, 4i)**.

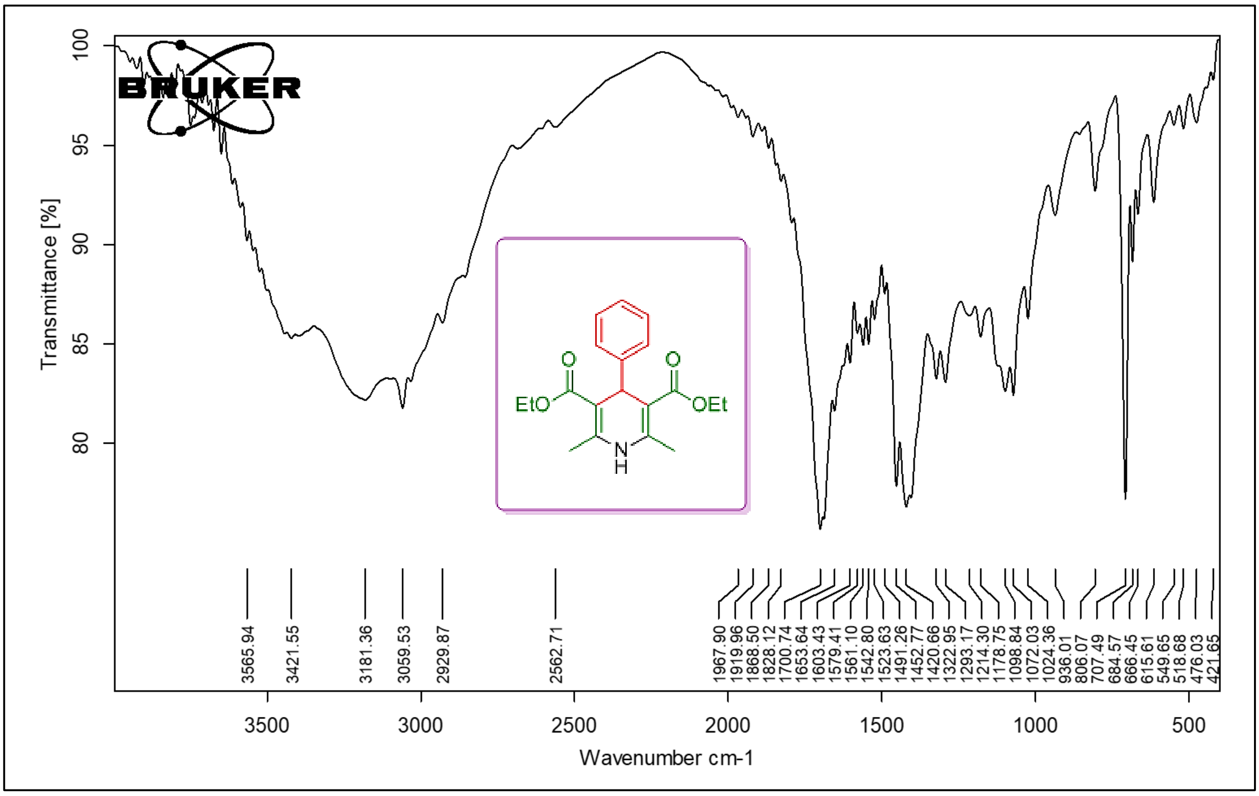

Figure S23. FTIR spectra of **(Table 1, 5a)**.

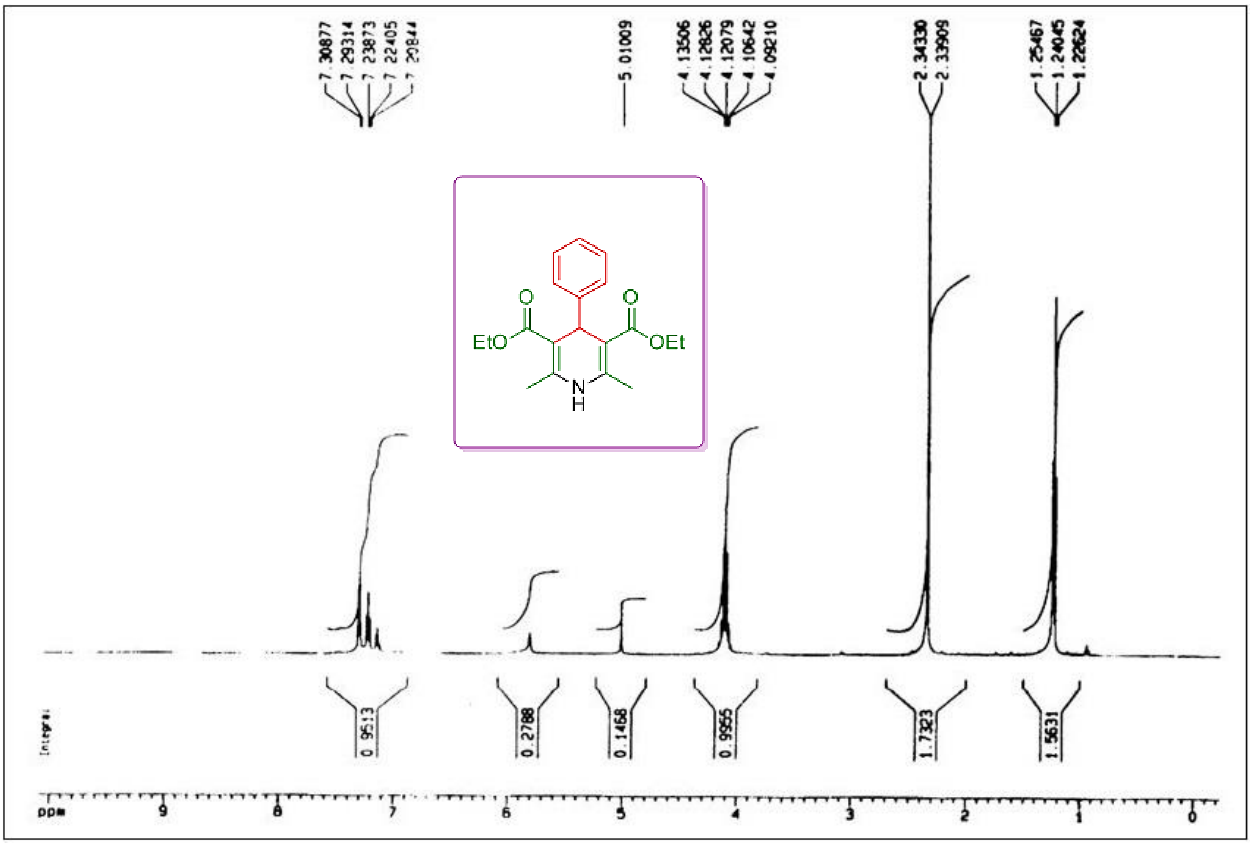

Figure S24. ¹H-NMR spectra of **(Table 1, 5a)**.

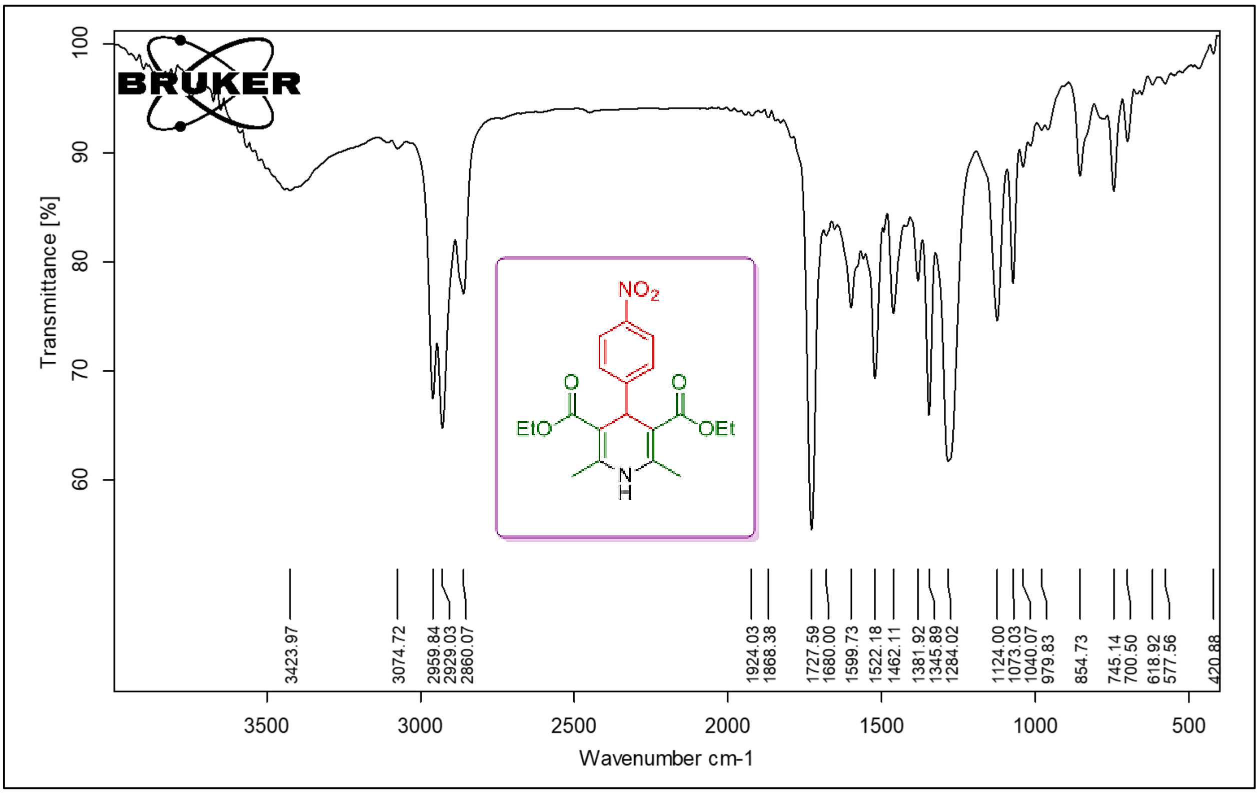

Figure S25. FTIR spectra of **(Table 1, 5b)**.

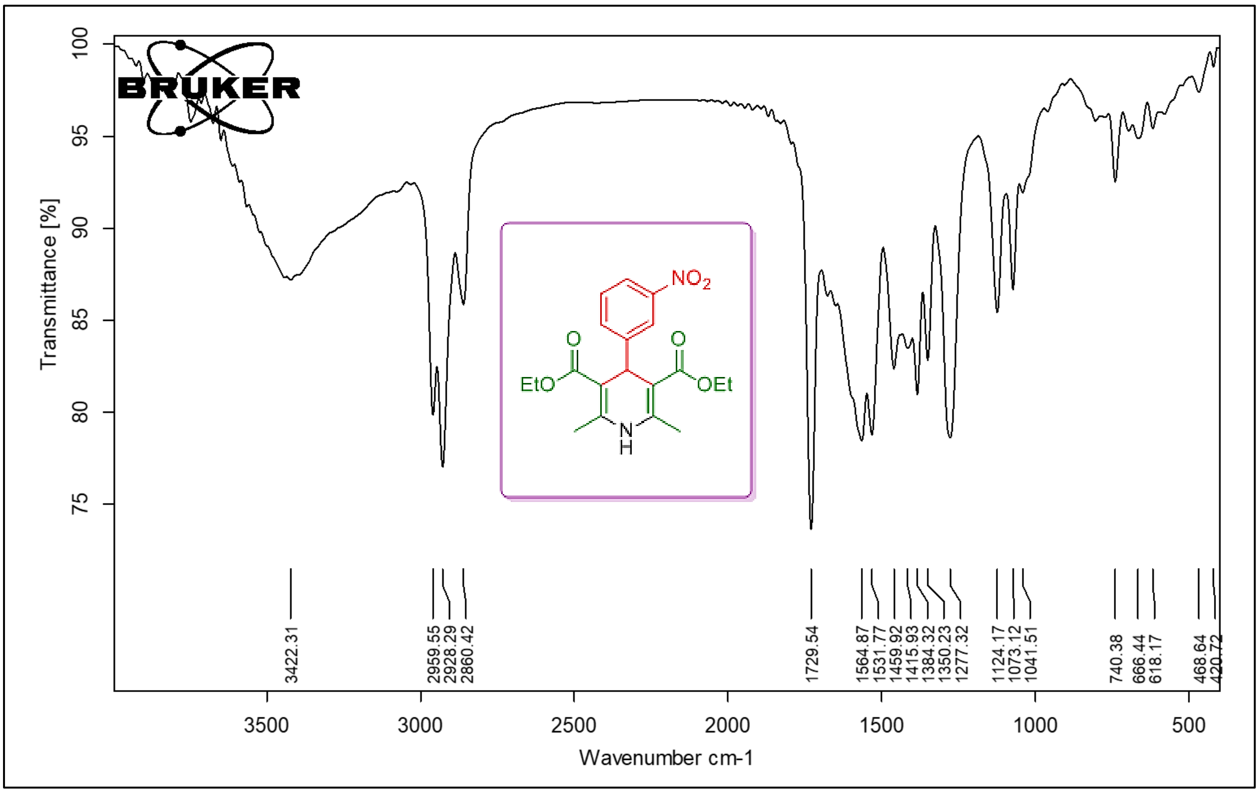

Figure S26. FTIR spectra of **(Table 1, 5c)**.

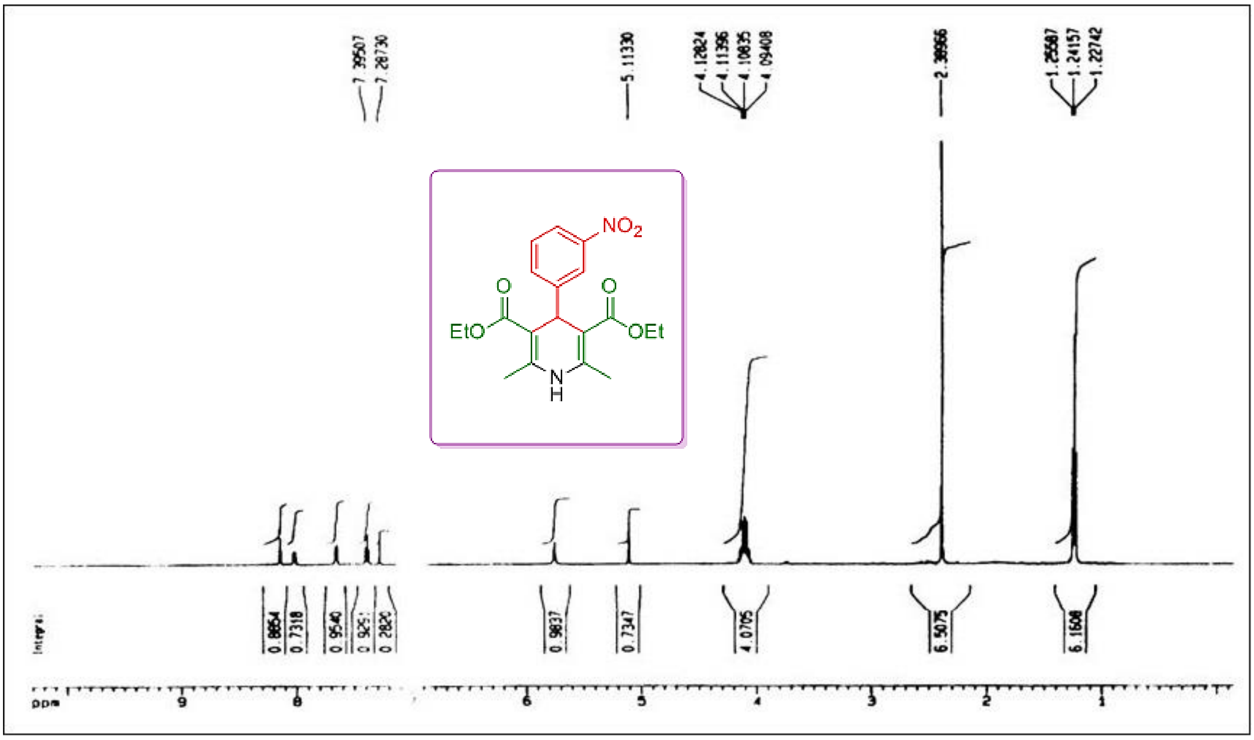

Figure S27. ¹H-NMR spectra of **(Table 1, 5c)**.

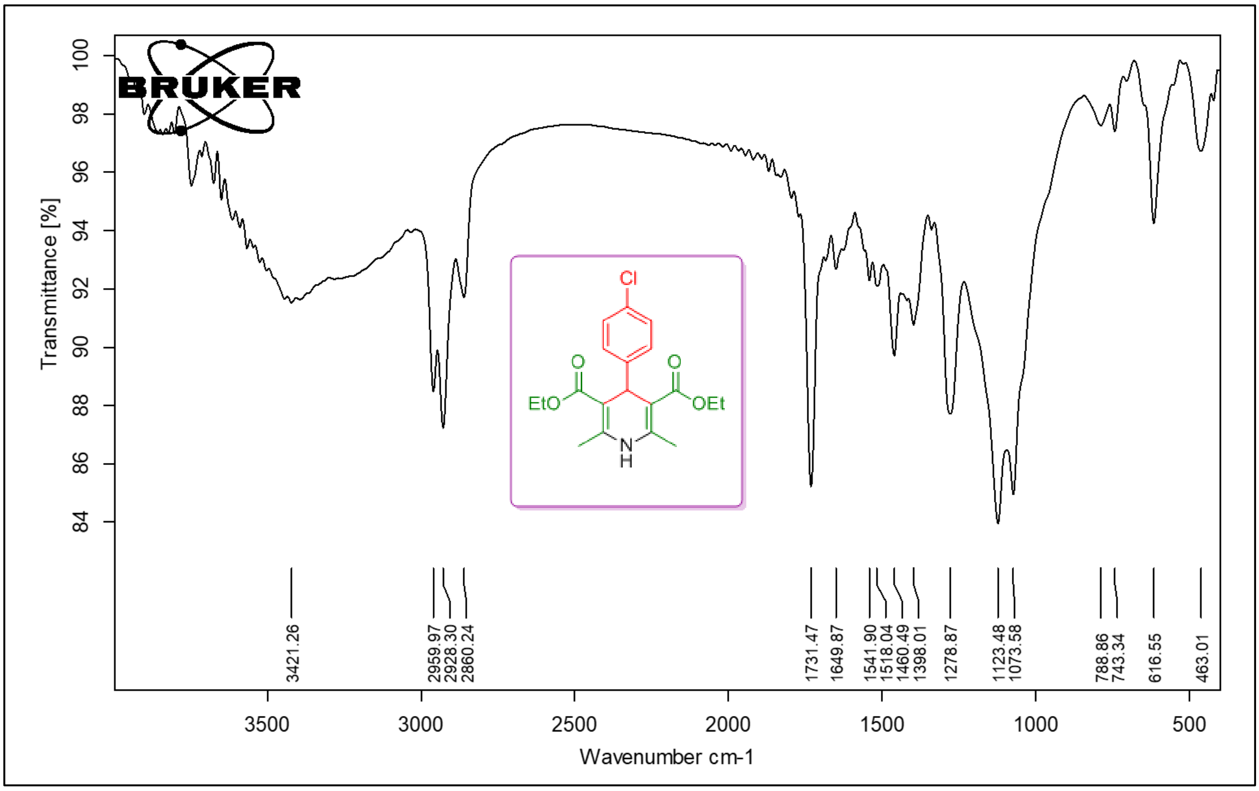

Figure S28. FTIR spectra of **(Table 1, 5d)**.

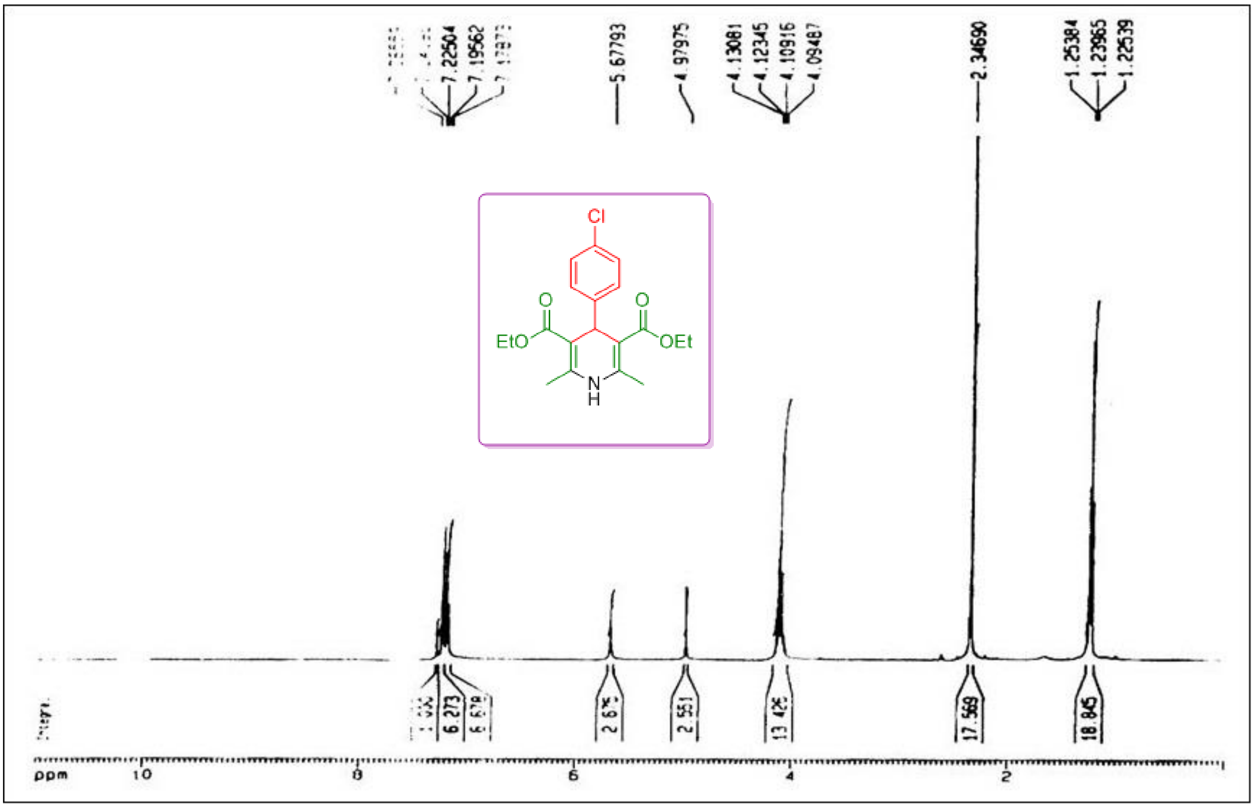

Figure S29. ^{1}H-NMR spectra of **(Table 1, 5d)**.

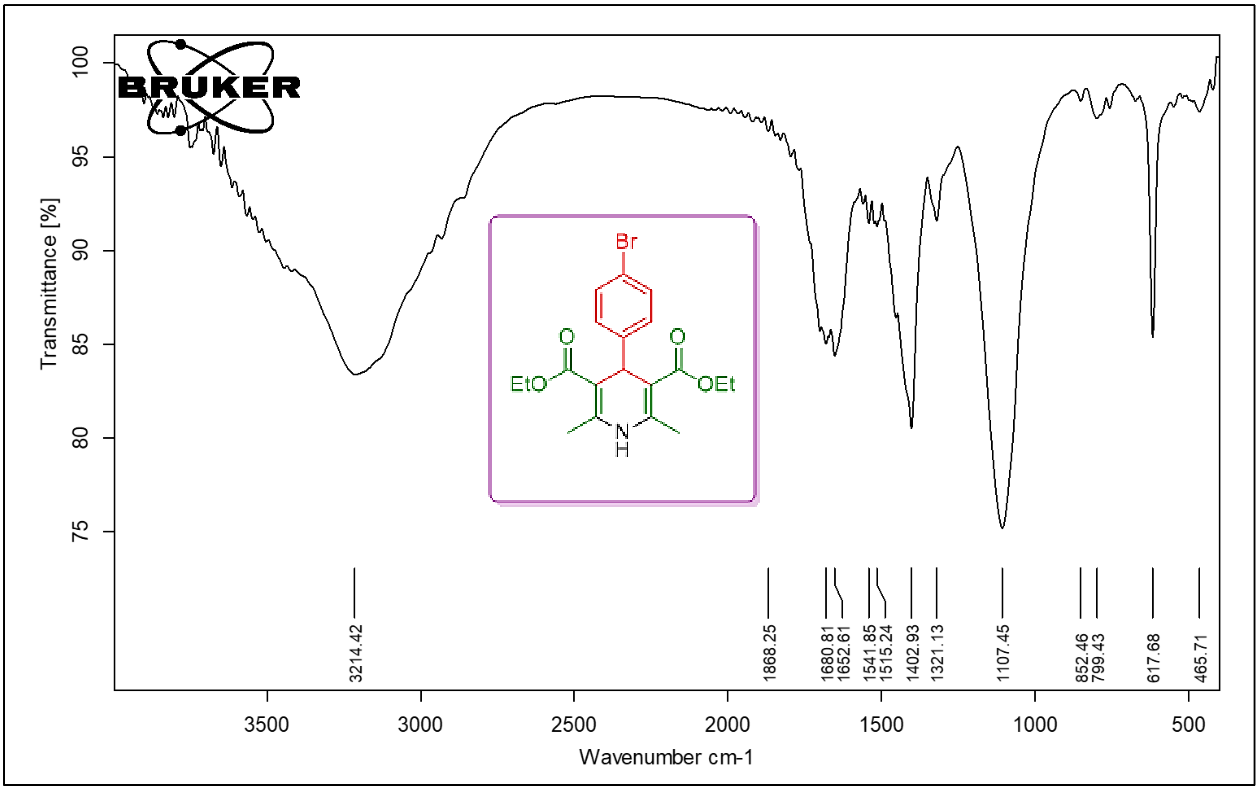

Figure S30. FTIR spectra of **(Table 1, 5e)**.

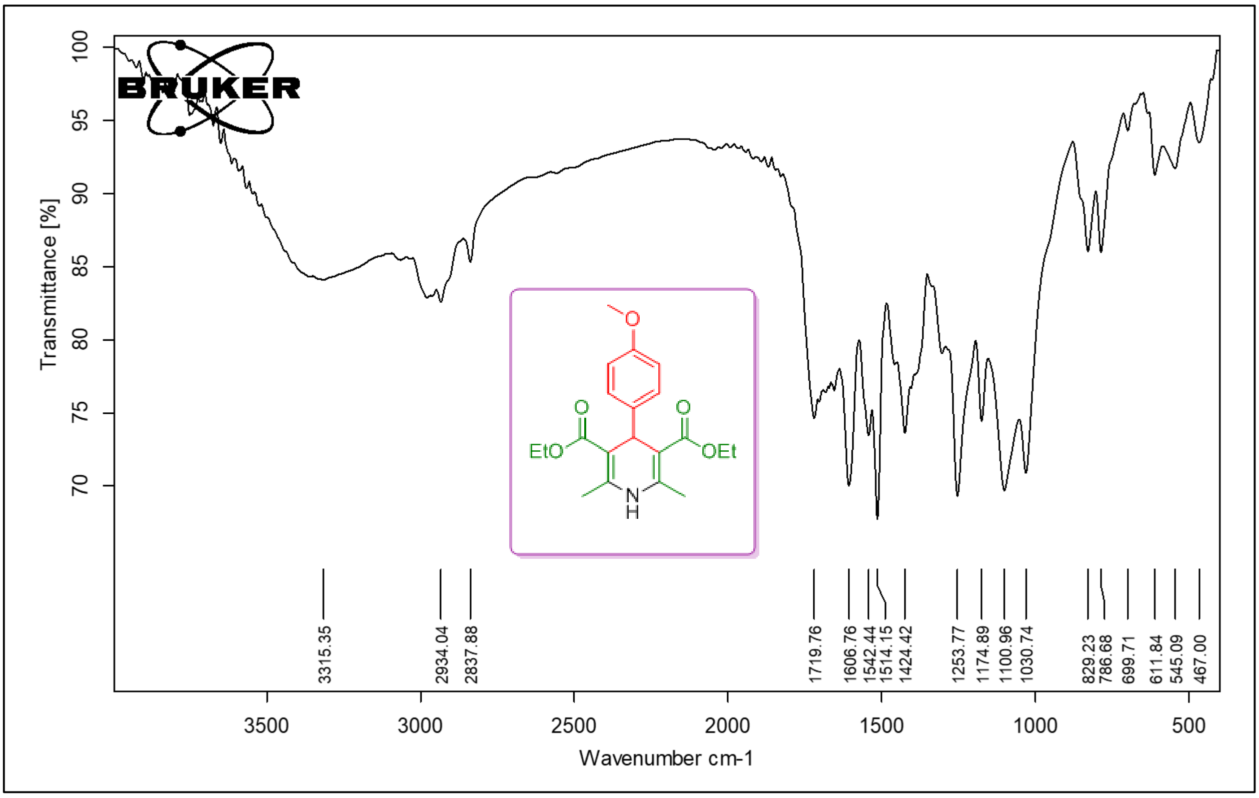

Figure S31. FTIR spectra of **(Table 1, 5f)**.

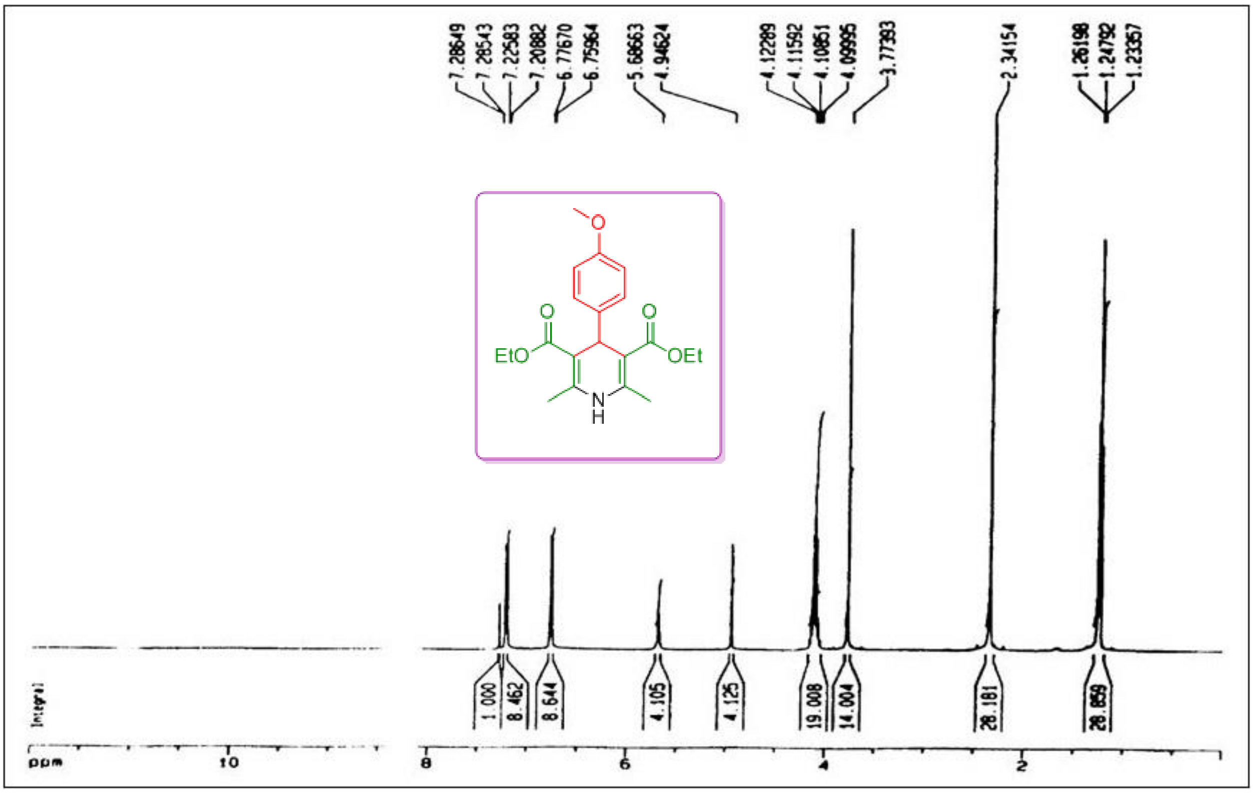

Figure S32. ^{1}H-NMR spectra of **(Table 1, 5f)**.

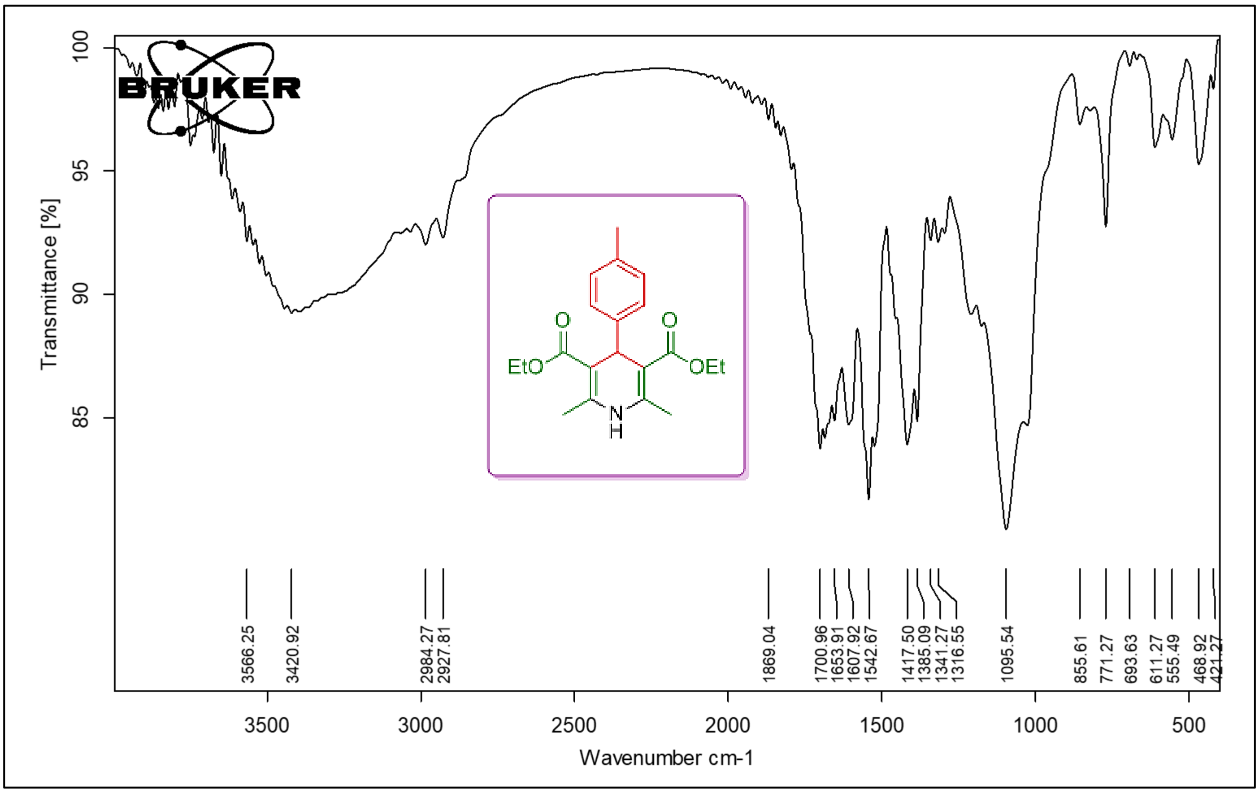

Figure S33. FTIR spectra of **(Table 1, 5g)**.

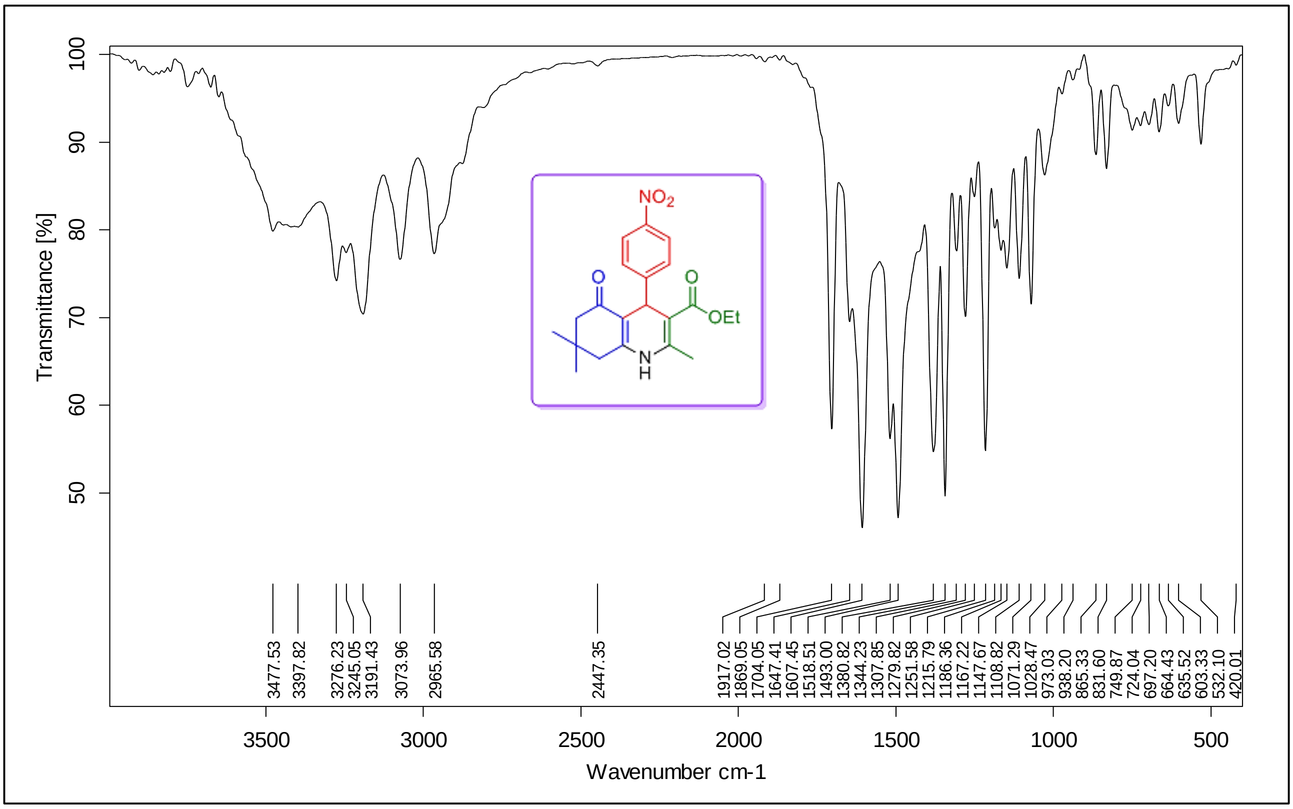

Figure S34. FTIR spectra of **(Table 2, 6b)**.

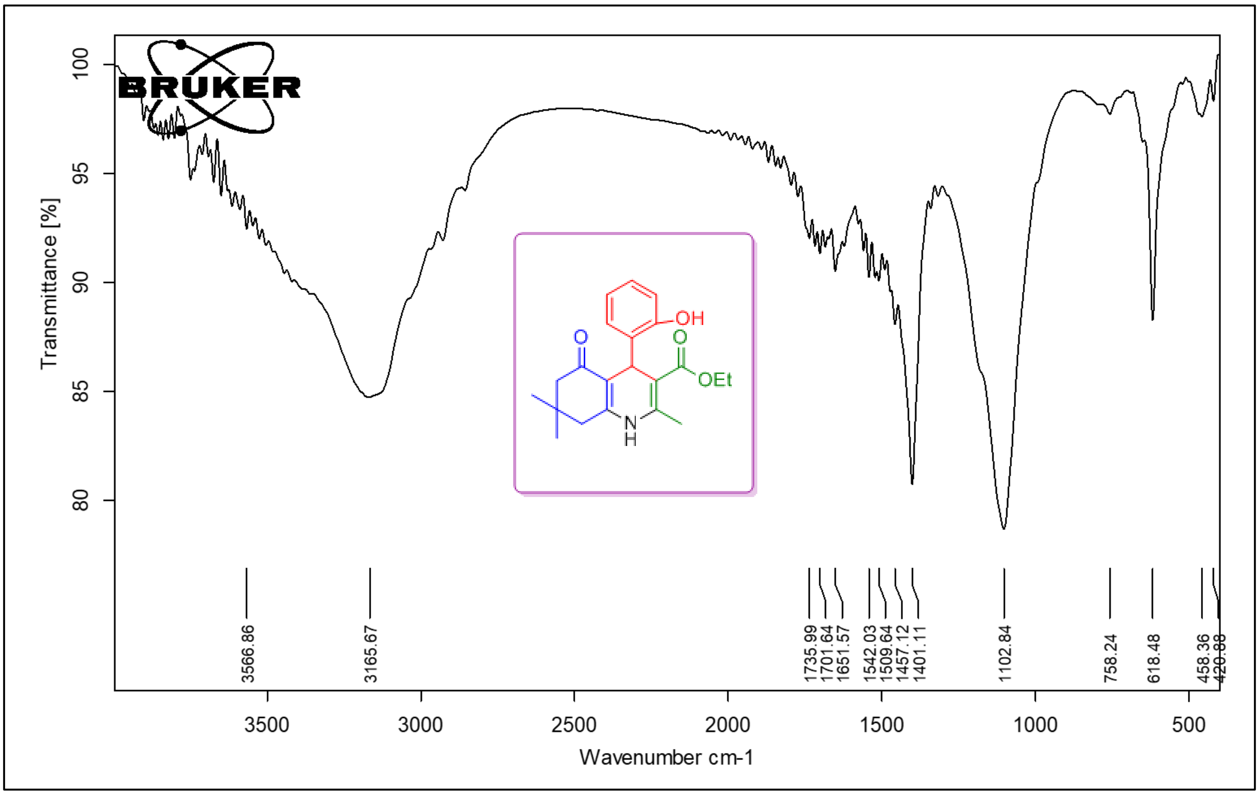

Figure S35. FTIR spectra of **(Table 2, 6c)**.

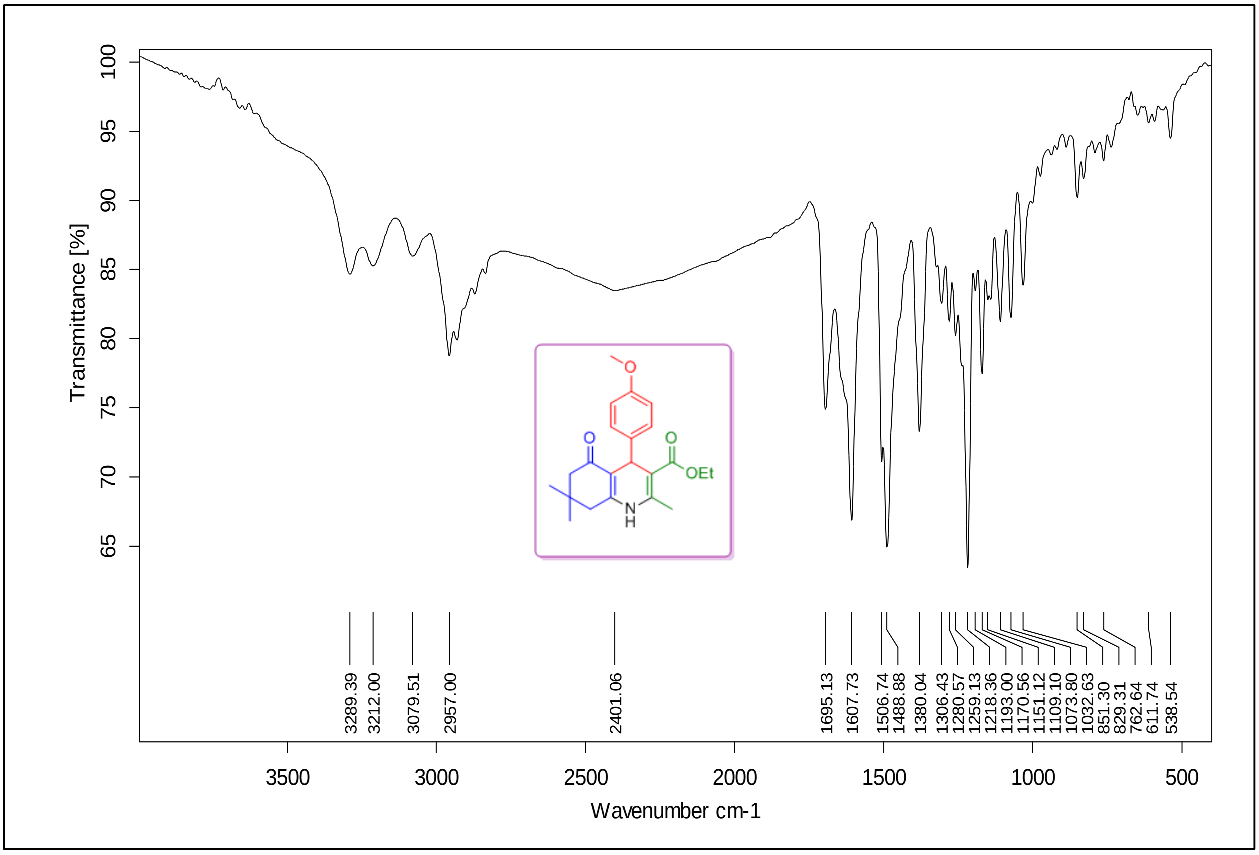

Figure S36. FTIR spectra of **(Table 2, 6d)**.

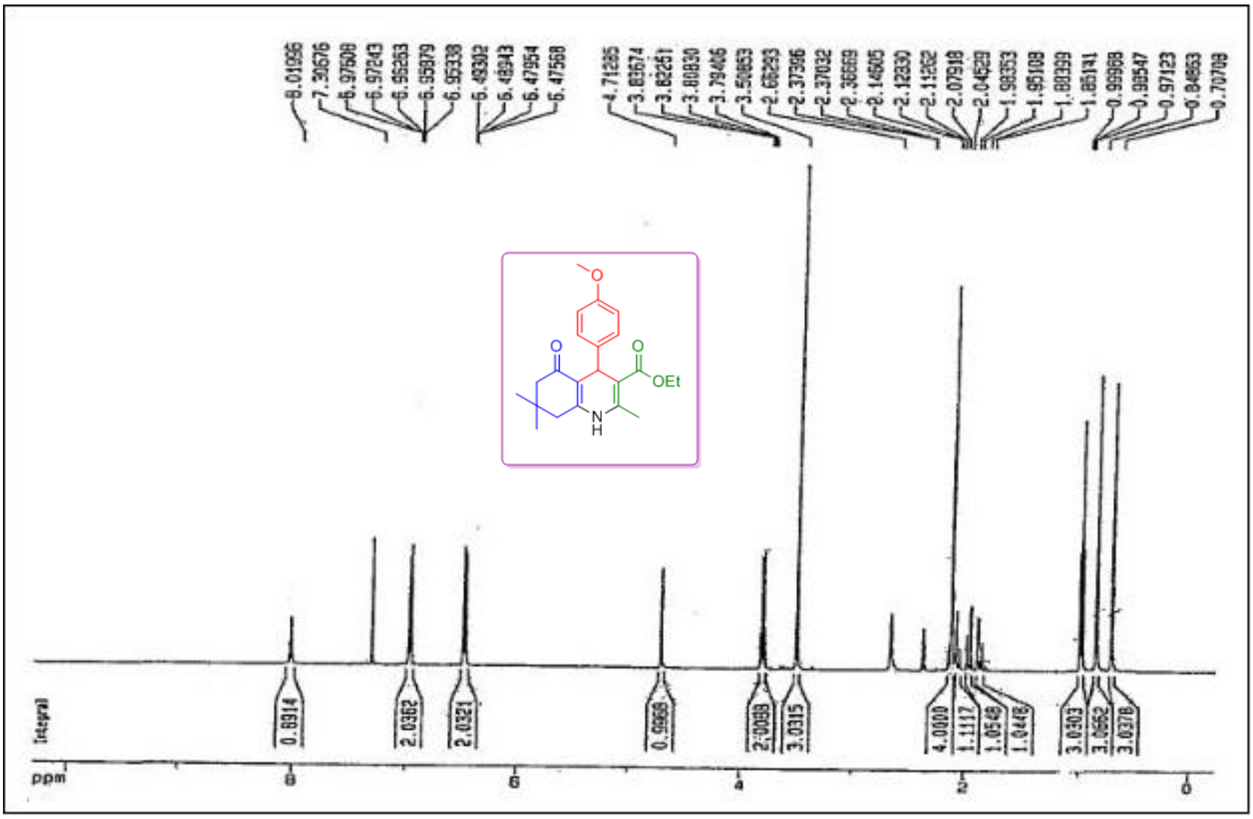

Figure S37. ^{1}H-NMR spectra of **(Table 2, 6d)**.

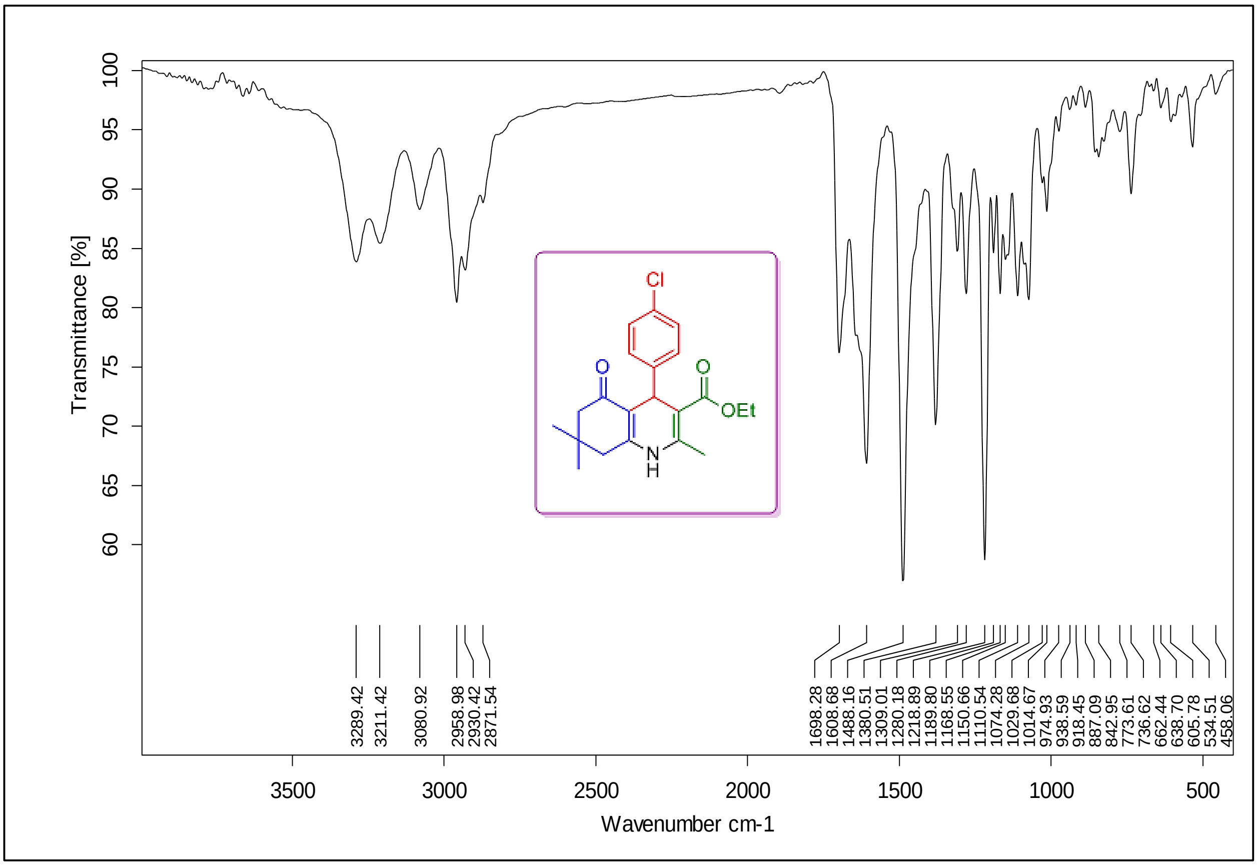

Figure S38. FTIR spectra of **(Table 2, 6e)**.

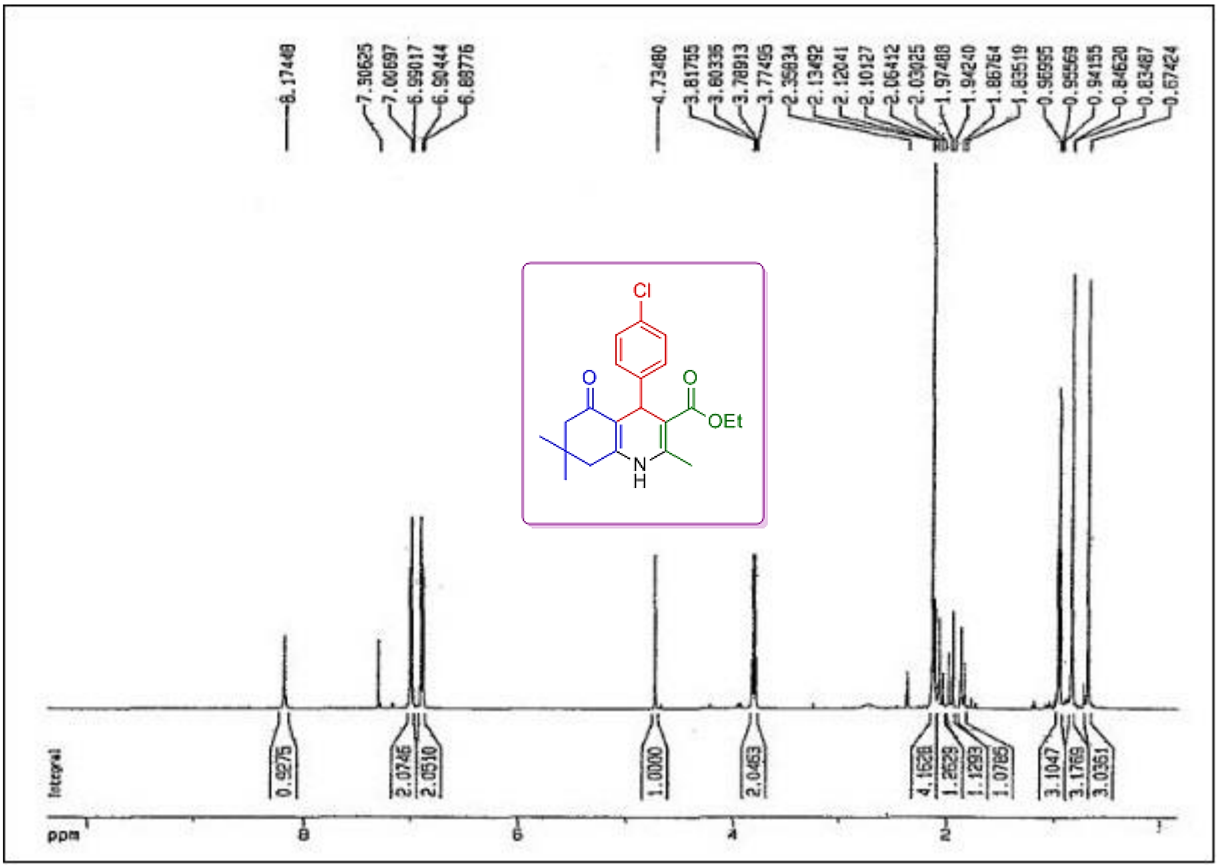

Figure S39. ^{1}H-NMR spectra of **(Table 2, 6e)**.

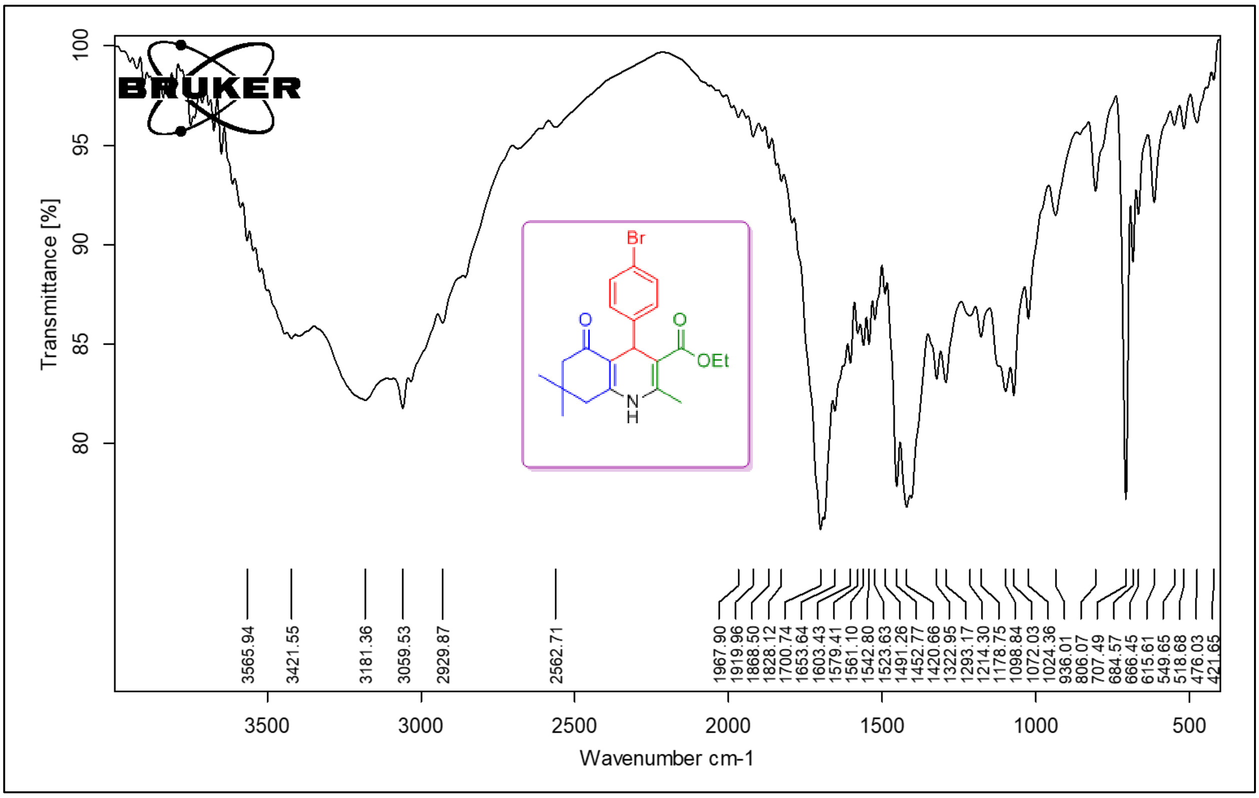

Figure S40. FTIR spectra of **(Table 2, 6f)**.

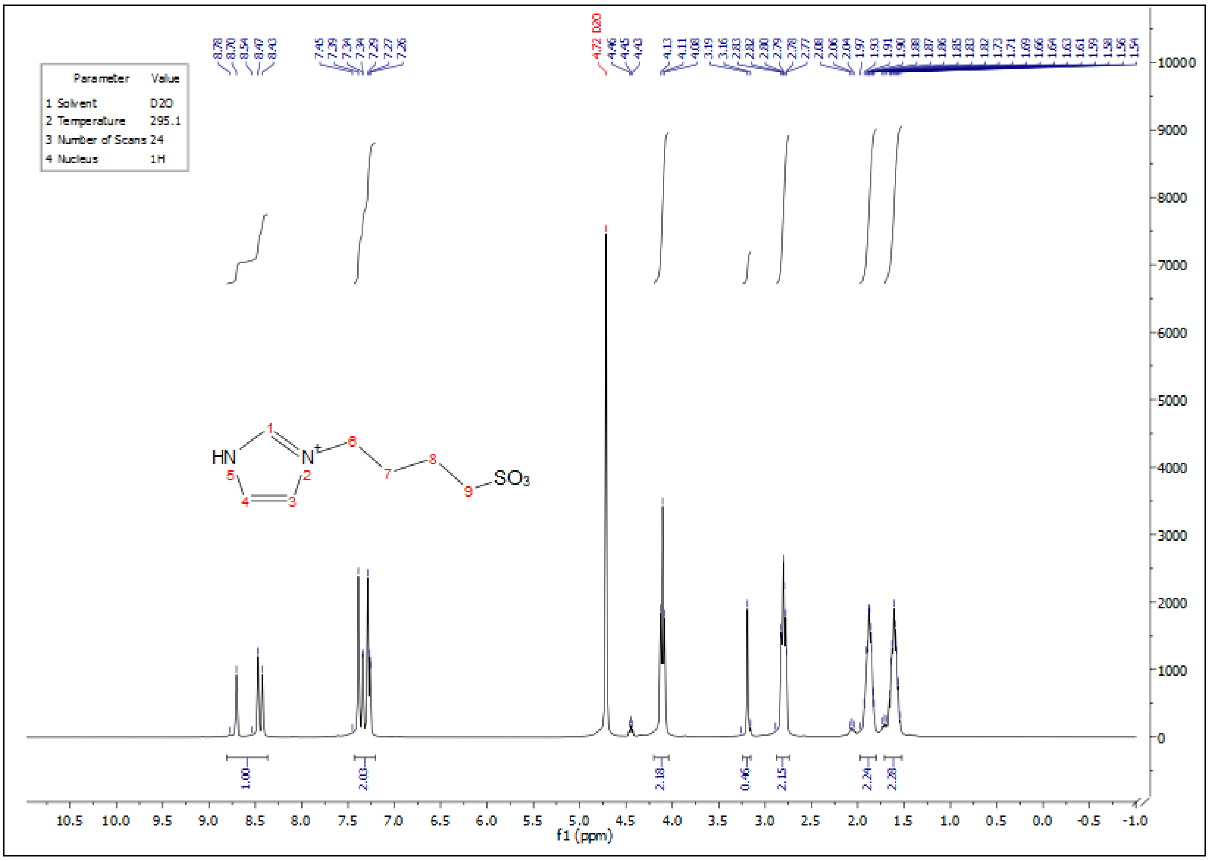

Figure S41. ^{1}H-NMR spectra of 3-(n-butanesulfonate)-imidazole.

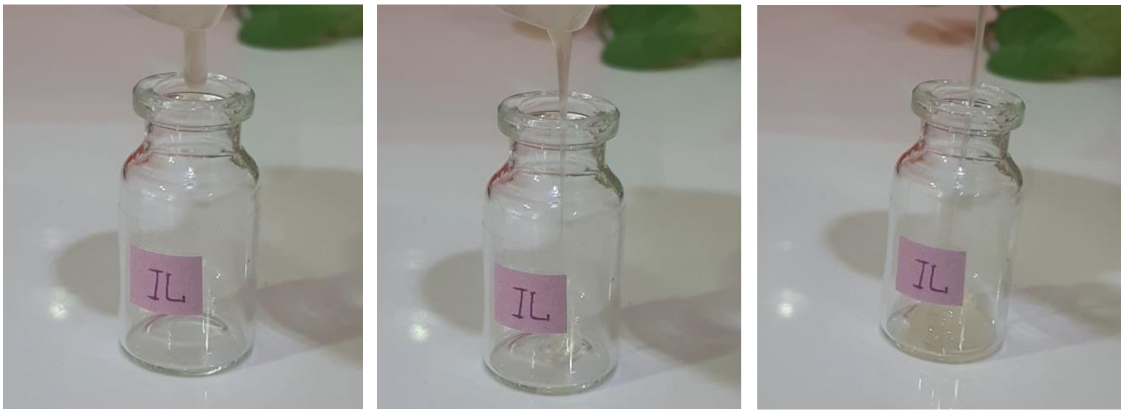

Figure S42. 3-(n-butanesulfonate)-imidazole.
